# Fatigue Failure Criteria of Asphalt Binders and Asphalt Mixtures: A Comprehensive Review

**DOI:** 10.3390/ma18143267

**Published:** 2025-07-10

**Authors:** Shizhan Xu, Zhigang Zhao, Honglei Wang, Chenguang Wan, Xiaofeng Wang, Zhenjun Wang, Xuanrui Zhang

**Affiliations:** 1 College of Civil Engineering, Zhengzhou University, Zhengzhou 450001, China; xushizhan@zzu.edu.cn (S.X.); 13623839637@163.com (Z.Z.); 2 Research and Development Center of Transport Industry of Technologies, Materials and Equipment of Highway Construction and Maintenance, Zhengzhou 450000, China; cadx0688@163.com (X.W.); zhang.xuanrui@outlook.com (X.Z.); 3 Henan Provincial Key Laboratory of Solid Waste Material Recycling in Road Engineering, Zhengzhou 450000, China; 4 Henan Zhonggong Design & Research Group Co., Ltd., Zhengzhou 450000, China; 5 School of Materials Science and Engineering, Chang’an University, Xi’an 710061, China; zjwang@chd.edu.cn; 6 School of Civil and Environmental Engineering, University of Technology Sydney, Sydney, NSW 2007, Australia

**Keywords:** fatigue test method, failure criteria, fatigue approach, VECD model, hot mixture asphalt

## Abstract

This study presents a systematic review of fatigue analysis methodologies and failure criteria for asphalt binders and mixtures employed in various cyclic fatigue testing configurations. The investigation focuses on two principal predictive approaches: phenomenological models and mechanistic frameworks, which are commonly utilized to forecast asphalt pavement fatigue life based on experimental data from different fatigue tests. A critical evaluation is conducted on the diverse failure criteria integrated within these analytical approaches, with particular emphasis on their respective merits and limitations. The current research findings reveal a notable absence of consensus regarding the precise definition of the fatigue failure criteria for asphalt materials. Furthermore, critical parameters including accuracy assessment, reliability verification, and sensitivity analysis of these failure criteria are identified as requiring enhanced research attention. This review recommends specific fatigue failure criteria classified according to fatigue testing methods and material types. This comprehensive analysis of fatigue failure mechanisms in asphalt composites aims to inform strategic refinements for future research trajectories and enhance durability-oriented pavement design practices.

## 1. Introduction

The phenomenon of fatigue gained significant research attention following its initial observation in the 19th century, when cyclic loading induced fatigue cracks in European steel structures such as bridges and railroads. Pioneering research on fatigue behavior in hot mix asphalt (HMA) was initiated at Nottingham University during the 1950s [1]. Fatigue cracking—manifesting as bottom-up or top-down failure—constitutes a primary distress mechanism in asphalt mixtures. This phenomenon results from progressive damage accumulation under repeated traffic loading and extreme climatic variations (e.g., thermal cycling, precipitation, or solar radiation), ultimately evolving into macroscale cracks over time [2,3,4,5,6,7,8,9]. These fatigue cracks occur due to the tensile and compressive strains that develop in reverse directions at the surface and bottom regions of asphalt pavement layers under repeated axle loads. These cracks manifest as hexagonal patterns, longitudinal fissures, and alligator cracking on pavement surfaces, which can lead to a decrease in the driving quality and fuel economy by increasing the roughness of the road, and provide channels for water intrusion, especially in the wheel paths, causing the rapid deterioration of the pavement system and increasing maintenance costs [10,11,12]. In recent decades, fatigue cracking has become a major concern in pavement engineering due to its significant impact on pavement service life. Methodical investigation of fatigue cracking is imperative to underpin robust structural design paradigms, particularly with the deployment of advanced material technologies including highly polymerized asphalt binders (HPABs), high-modulus asphalt binders (HMABs), warm-mix asphalt (WMA) modifiers, and reclaimed asphalt pavement (RAP) integration.

Despite extensive investigations into asphalt binder and mixture fatigue behavior over the last few decades [13,14,15,16,17,18,19,20], the underlying failure mechanisms remain incompletely characterized. The laboratory-based characterization of asphalt mixture fatigue employs multiple methodologies: phenomenological frameworks [21,22], mechanistic principles, energy dissipation analyses, and artificial neural network (ANN) modeling. Based on the different test methods mentioned above, both domestic and international scholars have conducted extensive research to define numerous fatigue models, aiming to accurately predict the service life of asphalt pavements and guide the actual structural design of the pavement. However, existing research efforts have predominantly centered on the development of the models themselves, whereas the definition of the fatigue failure criteria—being of critical importance for fatigue performance prediction models—has been largely overlooked. The fatigue failure criterion directly determines the termination conditions of the fatigue test. When distinct fatigue failure criteria are employed, the extent of the fatigue damage incurred by the material at the conclusion of the test demonstrates significant variation. This phenomenon represents a key factor contributing to the substantial discrepancy in the fatigue life predictions of asphalt mixtures [23]. Also, different fatigue failure criteria can cause different theoretical foundations for predicting the fatigue performance of the mixtures. Wang [24] contends that scientifically validated laboratory testing protocols, efficient data analysis methodologies, and material-specific fatigue failure criteria constitute the three indispensable pillars for evaluating and predicting the fatigue performance of asphalt materials. To ensure the reliability of test outcomes and establish a uniform foundation for any implementation framework, there is a critical need for an accurate, standardized, and consistent failure definition [7]. Consequently, the formulation of fatigue failure criteria in laboratory settings represents a critical research issue.

Currently, the academic community has not reached a consensus on the definition of the fatigue failure criteria for asphalt binders and mixtures. Typically, the failure criteria are used to determine when a material fails, that is, the number of cycles corresponding to failure independent of the load modes, test temperatures, and strain amplitudes. They are closely related to the critical stages of the fatigue testing process and the fatigue life prediction model itself. Gudipudi and Underwood [2] divided the failure criteria into experimental failure criteria and model failure criteria, which are, respectively, designated as the failure indicator and failure criterion in other scholarly works [25]. For example, the failure criterion in AASHTO TP 107-18 [26], namely the relationship between G^R^ and N_f_, is considered a model failure criterion. In contrast, the conventional failure criterion [27], where the modulus value decreases to 50% of its initial value, is considered an experimental failure criterion.

As previously detailed, the determination of the fatigue failure criteria is closely related to the fatigue testing methods and life prediction models for asphalt binders and mixtures. Therefore, the objective of this work is to systematically elaborate on the fatigue testing methods and to summarize the failure criteria based on these methods and the established life prediction models. This endeavor aids in enhancing the reliability and accuracy of pavement performance predictions, facilitates the refinement or redefinition of the failure criteria for subsequent studies, and thus informs the durability-oriented design of in-field asphalt pavements.

## 2. Fatigue Test Methods

In laboratory settings, HMA fatigue life is determined by applying fatigue loading at differing initial strain or stress levels. To predict the field fatigue performance of asphalt pavements, laboratory test results are processed using shift factors, which integrate key long-term influences like aging, healing, traffic load spectra, thermal cycling, UV exposure, and moisture degradation. The shift factor may amplify laboratory-derived fatigue life estimates by a factor of 15 to 20 times [7]. Researchers have developed diverse fatigue testing methodologies with distinct protocols to evaluate HMA fatigue life over recent decades. The fatigue life assessment of HMA has historically utilized diverse laboratory methodologies, each employing unique experimental protocols. These tests are systematically classified according to two fundamental criteria: (1) loading mode, encompassing flexural bending, uniaxial direct tension/compression, and diametral loading configurations; and (2) stress–strain distribution, differentiating homogeneous specimens from non-homogeneous field-simulated approaches across the documented literature [7].

**Figure 1:**
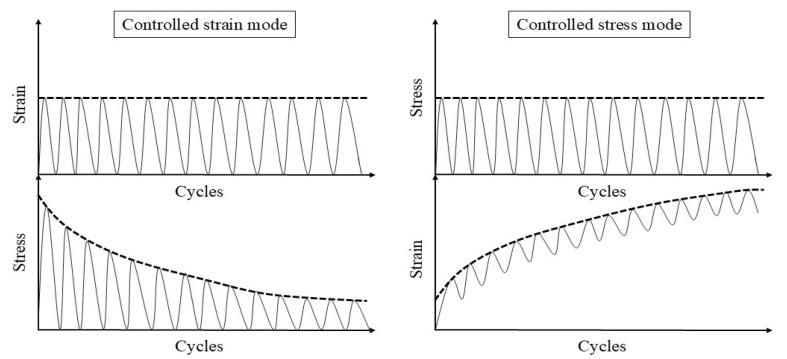
Contrasting stress–strain-cycle evolution in strain- vs. stress-controlled fatigue testing.

**Figure 2:**
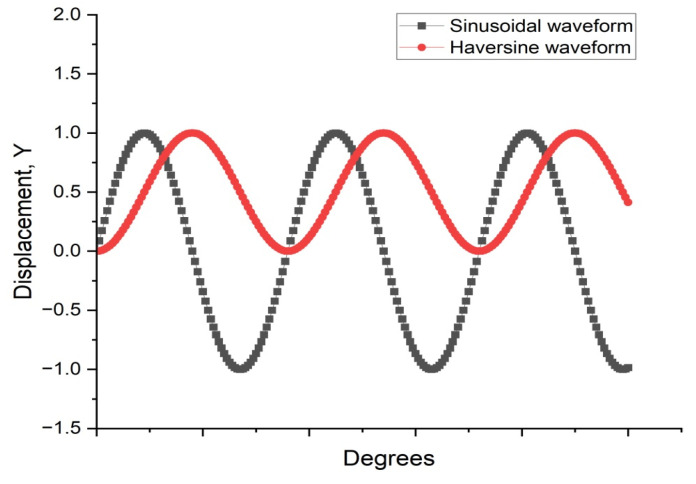
Illustration of haversine waveform relative to sinusoidal waveform.

Fatigue testing outcomes exhibit significant dependence on applied loading modes and waveform configurations under laboratory conditions. Four principal testing modes exist for hot mix asphalt fatigue evaluation: (i) load-controlled (stress), (ii) displacement-controlled (strain), (iii) energy-controlled [28,29], and (iv) hybrid methodologies [30]. Among these, stress- and strain-controlled approaches represent the predominant experimental paradigms, while energy-based and hybrid techniques exhibit more limited adoption. For identical asphalt mixtures under comparable initial fatigue testing conditions, the fatigue life observed in stress-controlled testing is notably reduced compared to that measured under strain-controlled conditions. This difference arises because stress-controlled tests maintain a constant stress amplitude (peak and valley values) on the specimen. Consequently, as loading cycles accumulate, the strain within the specimen progressively increases, ultimately causing fracture. Conversely, strain-controlled tests maintain a constant strain amplitude at the specimen’s critical location (e.g., the bottom). In this mode, the stress response gradually diminishes with increasing load cycles, as illustrated in Figure 1. Therefore, in the strain-controlled mode, the specimen does not exhibit noticeable cracking by the end of the test. For the same type of HMA under the same initial conditions, the fatigue life under strain-controlled testing exceeds that under stress-controlled conditions by a factor of approximately 2 to 3 times [31,32]. The loading waveforms in fatigue cyclic tests include sinusoidal waveforms [33] and haversine waveforms [34,35] ($=\sin^{2}\left(degrees/2\right)$). As depicted in Figure 2, a fundamental distinction between sinusoidal and haversine waveforms lies in their loading directionality: the sinusoidal waveform generates bidirectional stress/strain/deflection (alternating tension-compression), making it suitable for inducing fatigue damage in beam specimens under oscillatory loading. In contrast, the latter waveform has only one direction, and in cyclic loading fatigue tests, it can induce bending deformation in beam specimens, resulting in both permanent deformation and fatigue damage. This is closer to the repeated axle loads experienced by actual road surfaces [36].

**Figure 3:**
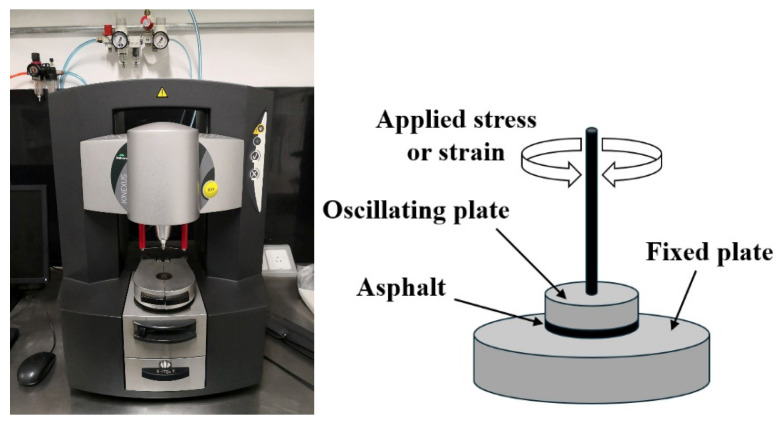
Schematic of the dynamic shear rheometer.

**Figure 4:**
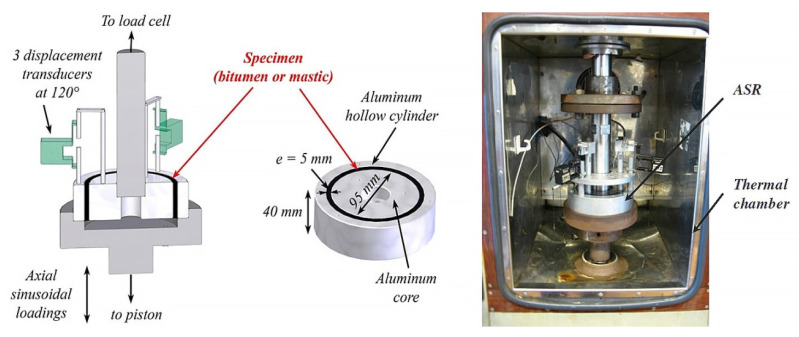
Diagrammatic representation of the annular shear rheometer [49].

While the dynamic shear rheometer (DSR), depicted in Figure 3, is utilized to characterize the dynamic shear properties of asphalt binders, it also serves as the key instrument for evaluating their fatigue performance via the Linear Amplitude Sweep (LAS) and Time Sweep (TS) tests [37]. Furthermore, a novel apparatus termed the annular shear rheometer (ASR) has been developed to characterize the fatigue behavior of bitumens and mastics, as illustrated in Figure 4.

**Figure 5:**
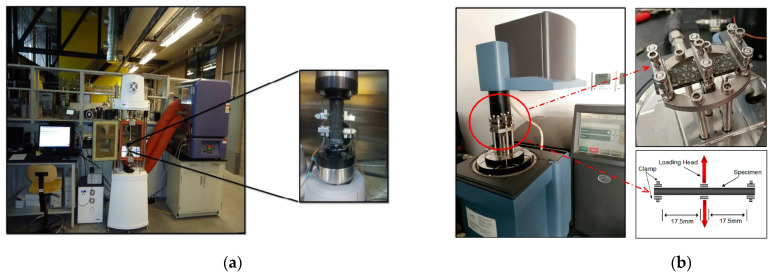
Fatigue test methods of FAM: (**a**) uniaxial fatigue testing configuration for FAM specimen [50]; and (**b**) dynamic mechanical analyzer (DMA) dual-cantilever bending clamp configuration [51].

**Figure 6:**
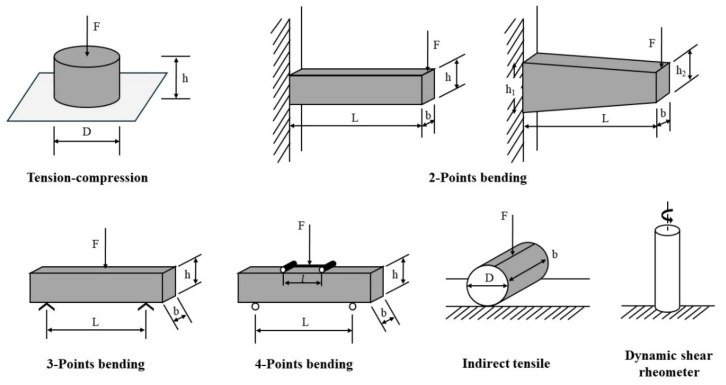
The fatigue test methods for AC.

Figure 5 and Figure 6 summarize the experimental methodologies employed for fatigue damage evaluation in fine aggregate matrix (FAM) and asphalt concrete (AC) mixtures, respectively. Fatigue performance may also be evaluated using other tests, such as the supported flexure test [7], double-edge notched test, overlay tester, dogbone tester, loaded wheel tester [38], disk compact tension (DCT) [39,40], semi-circular beam (SCB) I-FIT method [41,42], semi-circular beam (SCB) LTRC method [43,44], single-edge notched beam (SENB) [45,46], and double-edge notched prism (DENP) [47], along with the UGR-FACT (University of Granada-Fatigue Asphalt Cracking Test) method [9,48]. Table 1 summarizes the various testing methodologies employed to assess the fatigue life of both asphalt binders and mixtures.

**Table 1:** Loading modes and schematic diagrams of specimens under different fatigue tests.

Loading Mode/Test Method	Schematic Diagram	Material Type	References
Uniaxial compression/tension (UT/UC)	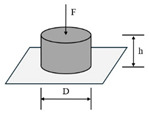	AC	[26,33,52,53]
Beam bending (BB)	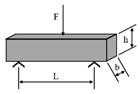 Three-point bending (3PB)	AC	[54,55]
	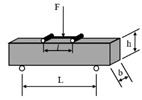 Four-point bending (4PB)		[34,35]
	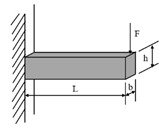 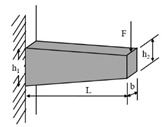 Two-point bending (2PB)		[56,57,58,59]
Indirect tensile (IDT)	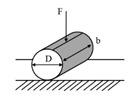	AC	[60,61,62,63]
Dynamic shearing (DS)	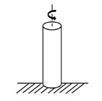	AC	[64,65,66]
Semi-circular bending (SCB)	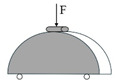	AC	[41,67]
Dynamic mechanical analyzer (DMA)	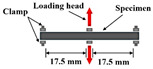	FAM	[51,68]
Annular shear rheometer (ASR)	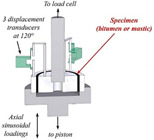	Asphalt or mastic	[49,69,70]
Linear Amplitude Sweep (LAS)	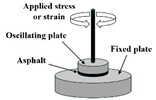	Asphalt	[71,72]

The aforementioned fatigue testing methods enable the evaluation of fatigue performance in both asphalt binders and mixtures. To predict the fatigue life of asphalt pavements, various fatigue analysis approaches have been established based on test data interpretation. The principal categories encompass phenomenological models, mechanistic principles, and artificial neural network techniques. To define fatigue performance within regions of pronounced material behavioral transition, failure criteria are employed as essential reference points [7,73]. These criteria directly determine the termination conditions of fatigue tests and the degree of fatigue damage to the material. Failure criteria are closely associated with fatigue approaches and are divided into experimental failure criteria and model failure criteria [2].

Since Hveem [74] discovered the fatigue failure of asphalt pavements and quantified the correlation between tensile strain/stress at the asphalt layer base and load cycle accumulation upon pavement cracking, the phenomenological approach, sometimes called the traditional approach or classical approach, has become one of the main research approaches for evaluating the fatigue damage characteristics and life prediction of asphalt mixtures. In general, the mechanistic approaches include fracture mechanics and the viscoelastic continuum damage (VECD) theory [7,75,76]. In addition, the viscoelastic fracture mechanics (VEFM) method, developed by combining the two concepts in the literature, has also been applied to the fatigue life modeling framework for asphalt–filler composites [77]. Recently, artificial neural network (ANN) approaches have been applied to characterize and predict the fatigue performance and behavior of materials due to their adaptability and learning advantages, replacing conventional approaches [78,79,80,81,82,83]. Although some researchers focus on the ANN approach for predicting the fatigue life of asphalt mixtures, there is currently a lack of a perfect computational model that can accurately predict fatigue life under various complex conditions. The ANN approach serves only as an auxiliary prediction tool, as it is based on the development of algorithms and relies heavily on large amounts of data.

The following section of this paper will provide a review of fatigue approaches, models, and failure criteria, aiming to promote their application, summarize the recent progress in this research field, and provide better inspiration for road engineers.

## 3. Failure Criteria for Fatigue Approaches

### 3.1. Phenomenological Approaches

Within the phenomenological frameworks, the asphalt mixture’s fatigue behavior is typically characterized by the initial stress and strain relationship [7]. The phenomenological models are established based on the analysis of laboratory fatigue test results using the phenomenological approaches, which are the earliest and simplest models used to define the fatigue life of HMA. Except for the traditional phenomenological fatigue models, sometimes called the basic fatigue models, energy-based models or dissipated energy models are also considered phenomenological models, since energy-based models involve the inductive or regression analysis of experimental data, where the dissipated energy parameter remains a phenomenological measure. It cannot distinguish between the dissipated energy from fatigue damage and the viscoelastic dissipation inherent in the mixture itself, nor can it detail the finer aspects of energy transformation. Thus, it cannot reveal the entire process of damage occurrence and development from the fundamental viscoelastic properties of the asphalt mixture.

#### 3.1.1. Failure Criteria of Basic Fatigue Models

Pioneering research in the 1960s by Monismith et al. [84] established correlations between tensile stress or strain at the base of asphalt layers and the number of load cycles to failure (commonly defined by a 50% reduction in the modulus/stiffness). This foundational work led to the development of the earliest predictive fatigue life models for pavement design and analysis by Monismith and Deacon [85] and Pell [86], formulated as Equations (1) and (2).

(1) $$N_{f}=a{\left(\frac{1}{\varepsilon_{t}}\right)}^{b}$$

(2) $$N_{f}=c{\left(\frac{1}{\sigma_{t}}\right)}^{d}$$

where $N_{f}$ is the number of cycles until fatigue failure; $\varepsilon_{t}$ is the strain level; $\sigma_{t}$ is the stress level, Pa; and coefficients *a*, *b*, *c*, and *d* are regression parameters.

Equations (1) and (2) characterize the fatigue life response of asphalt mixtures to varying strains (strain-controlled mode) and stresses (stress-controlled mode), respectively. Early research further established that tensile strain exhibits a stronger correlation with fatigue life than stress [86,87,88]. In 1972, scholars [89,90,91] recommended adding the mix stiffness to Equation (1), resulting in Equation (3). Later, Claessen et al. also verified that the addition of the asphalt stiffness is reasonable [92].

(3) $$N_{f}=a{\left(\frac{1}{\varepsilon_{t}}\right)}^{b}{\left(S_{mix}\right)}^{c}$$

where $N_{f}$ is the number of cycles to failure, $\varepsilon_{t}$ is the strain level, $S_{mix}$ is the mix stiffness of asphalt mixture, MPa, and parameters *a*, *b*, and *c* are calibrated using the recession analysis of experimental fatigue testing data.

**Figure 7:**
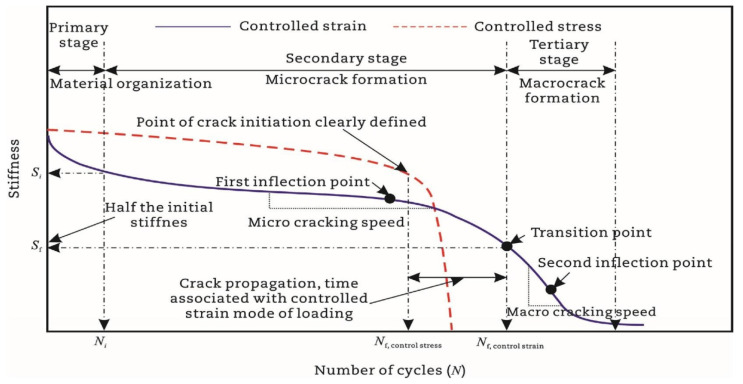
Variation in stiffness (complex modulus) as a function of load cycles under controlled-strain and controlled-stress testing [7].

Previous studies [52,93,94,95,96,97] report that the asphalt mixture stiffness modulus (e.g., complex modulus or phase angle) evolves through three distinct phases during fatigue testing: Phase I (adaptation), Phase II (quasi-stationary), and Phase III (failure), as schematized in Figure 7.

- Phase I (adaptation) exhibits rapid stiffness degradation. The two biasing phenomena, including heating caused by energy dissipation and binder thixotropy, can be interpreted for the sudden loss in stiffness [52]. When the test is paused at this stage, this loss of stiffness can be easily recovered;
- Phase II (quasi-stationary) exhibits progressive quasi-linear stiffness decay. Concurrently, microcrack nucleation emerges as the defining fatigue mechanism during this stage;
- Phase III (failure) is characterized by the catastrophic propagation of coalescence-induced macro-cracks beyond a critical damage threshold. The fatigue test cannot be considered homogenous anymore.

As scholars have delved deeper into the study of the fatigue life of asphalt mixtures, more potentially influencing factors have been incorporated into the models, such as the phase angle, temperature condition, rest period indicator, fracture properties (i.e., the fracture work density and the fracture energy), damage state of the asphalt mixture, and some internal parameters of asphalt binder and mixture properties [60,98,99,100,101,102,103,104,105,106,107,108].

(1) Stiffness Modulus Reduction Criteria

**Figure 8:**
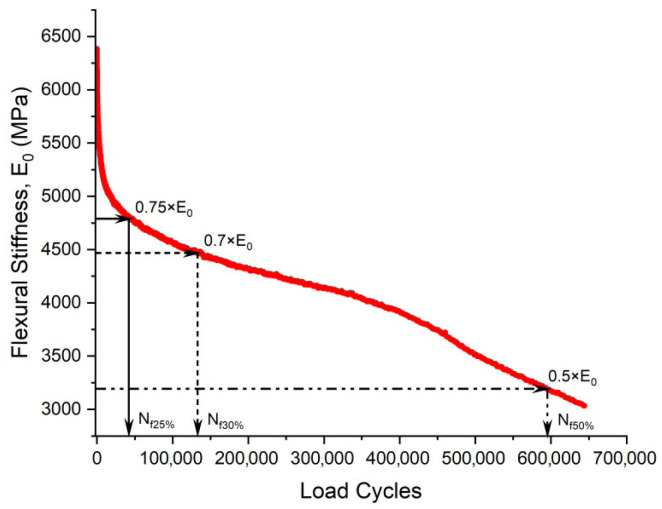
The schematic diagram of fatigue failure criteria at 25%, 30%, and 50% decrease in initial stiffness modulus.

Within the conventional fatigue testing of asphalt mixtures under the controlled-strain mode, the fatigue life is characterized by the load cycles required for the stiffness modulus to degrade to a predefined fraction of its initial value. The widely adopted termination criterion for this mode is a 50% reduction in the initial stiffness modulus, where testing ceases once the specimen’s modulus falls below half of its initial value [109,110]. This defines the $N_{f50\%}$ criterion, extensively utilized within the field [27,75,111,112,113]. Researchers also employ the $N_{f30\%}$ criterion, representing the load cycles required for a 30% stiffness reduction [97]. Di Benedetto et al. further recommended a failure threshold at a 25% stiffness decrease with thermal–thixotropic corrections [28]. Figure 8 provides a schematic representation of these three criteria applied to flexural beam fatigue tests.

Kim et al. [31,68] used the transition point, $N_{t}$, at the end of Phase II shown in Figure 7, to indicate the shift from microcracks to macro-cracks on the plot of the relationship between the stiffness loss caused by cumulative fatigue damage and the number of cycles in a typical controlled-strain fatigue test. The transition point is commonly used as a failure criterion to determine the fatigue life of asphalt mixtures. Additionally, it can be observed that in Figure 7, the transition point corresponds to exactly half the initial stiffness. Hence, to some extent, the criterion of the transition point is the same as with a 50% reduction in the initial stiffness modulus. Furthermore, based on the viscoelasticity and continuum damage theory, Lee [114,115] demonstrated that the 50% decrease in pseudostiffness was an effective criterion independent of the test conditions through uniaxial testing.

Conventional controlled-stress fatigue testing defines failure as either a specimen fracture or a 90% reduction in the initial stiffness modulus [31,116,117,118]. In the indirect tensile fatigue test, the fatigue failure point is defined as the number of load cycles when either specimen fracture occurs, or when the vertical displacement of the upper loading bar reaches 9 mm [119]. Certain researchers adopt a 100% increase in material strain (i.e., the doubling of the initial strain) as the fatigue failure criterion [98]. Sun et al. [120] proposed a fatigue failure criterion for in-service emulsified asphalt cold recycled mixtures through controlled-stress splitting fatigue tests, which is defined as follows: fatigue failure initiates at a 45% retention of the initial stiffness modulus and terminates upon further degradation to 45% of this failure–initiation modulus value. The fatigue failure criterion for laboratory-molded cold recycled asphalt mixture is defined as the beginning of fatigue failure when the stiffness modulus decreases to 35% of the initial modulus, and it ends when the specimen is completely destroyed. Moreover, Rowe et al. [121] challenge the applicability of the traditional 50% criterion for asphalt mixture fatigue assessment, citing evidence that failure manifests at a 35–65% residual initial stiffness modulus.

While flexural beam fatigue testing remains a traditional and widely adopted method for characterizing the fatigue behavior of asphalt mixtures, the inherent inhomogeneity of deformation within the beam during bending is often overlooked. This inhomogeneity leads to varying degrees of damage developing across different beam sections. Consequently, the stiffness modulus derived from fatigue test data inherently represents a weighted average value across the specimen [122]. Addressing this limitation, Abhijith and Narayan [123] introduced a novel fatigue failure criterion based on the evolution of the local modulus. They define the failure point as the cycle number at which the local modulus reaches zero, arguing that this provides a more physically significant alternative to the conventional criteria based on the global stiffness modulus reduction.

It should be emphasized that the stiffness modulus reduction criteria are primarily applicable to flexural beam fatigue tests, including three-point and four-point bending configurations. This limitation arises because, unlike flexural tests, uniaxial fatigue tests often do not exhibit a significant modulus decrease at failure. Crucially, the extent of modulus degradation in asphalt mixtures at fatigue failure during uniaxial testing is contingent upon the material’s initial modulus and the test temperature [124,125].

The traditional fatigue failure criterion faces criticism for its arbitrariness in defining failure, stemming from an absence of a theoretical foundation or physical basis. This limitation hinders consistent fatigue life predictions and overlooks the reversible biasing effects occurring in phase I [97,126,127,128,129,130]. Consequently, establishing more scientifically grounded failure criteria is imperative for achieving enhanced accuracy in predicting the fatigue failure life of asphalt mixtures.

(2) Phase Angle Criterion

The phase angle serves as a fundamental mechanical parameter for characterizing the viscoelastic response of asphalt mixtures under dynamic loading conditions. Specifically, the phase angle is the phase difference between the stress (or stress wave) and the strain (or strain wave). The calculation equation for the phase angle of asphalt mixture is shown in Equation (4).

(4) $$\phi =\left(\frac{∆t}{t_{p}}\right)\times 360{}^{\circ}=2\pi f\left(∆t\right)$$

where ϕ is the phase angle, in degrees, ∆t is the time difference between strain lagging behind stress within the same cycle, s, $t_{p}$ is the complete loading period of the stress or strain, s, and f is the loading frequency, Hz.

In perfectly elastic materials, stress and strain occur in phase (0° phase angle), whereas purely viscous materials exhibit a 90° phase difference. Viscoelastic materials demonstrate intermediate behavior with phase angles between 0° and 90°. This angle serves as an indicator of viscoelastic properties: higher values signify viscous dominance, while lower values reflect elastic dominance. Temperature dependence has been established for this parameter, with typical failure ranges observed between 10° and 50° [131].

**Figure 9:**
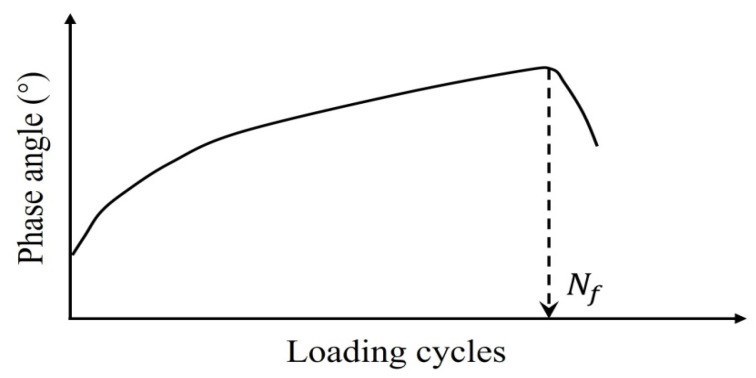
Variation in phase angle versus the loading cycles.

Studies [19,31,132] identify the peak in the phase angle versus the loading cycles (Figure 9) as a fatigue failure indicator for asphalt mixtures—termed the phase angle criterion. However, this criterion cannot predict the phase angle evolution in continuum damage models (e.g., VECD), and thus serves only to define rather than predict failure [133]. Consequently, it remains a fundamentally experimental failure metric. Shen and Lu’s analysis of binder/mixture fatigue data [128] revealed inconsistent correlations under strain-controlled loading for binders. Additionally, stress-controlled mixtures often lack discernible phase angle peaks.

(3) Fitting Change Point Criterion

**Figure 10:**
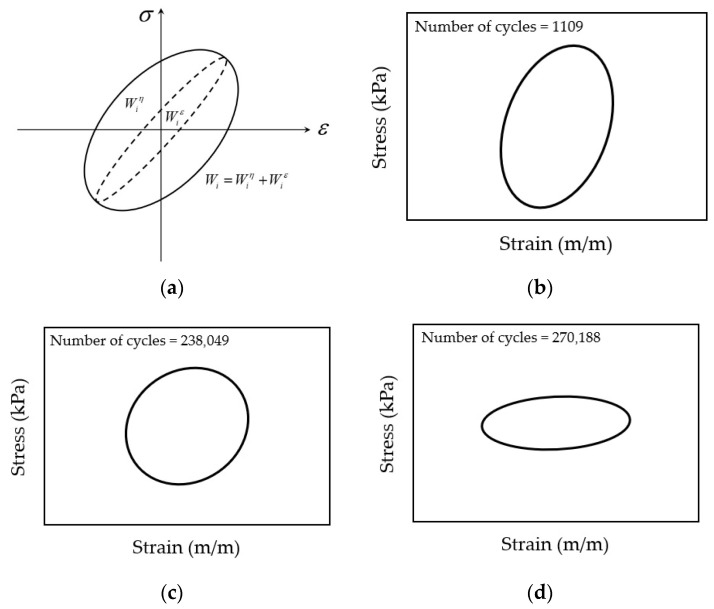
Viscoelastic properties: (**a**) stress versus strain relationship (hysteresis loops); (**b**,**c**) hysteresis loops at different number of cycles; and (**d**) distorted hysteresis loop.

The viscoelastic nature of asphalt mixtures prevents stress–strain curve superposition during cyclic loading, generating hysteresis loops (Figure 10a). Fatigue life determination occurs at the onset of loop distortion [134]. The distortion process of the hysteresis loop is shown in Figure 10b–d.

**Figure 11:**
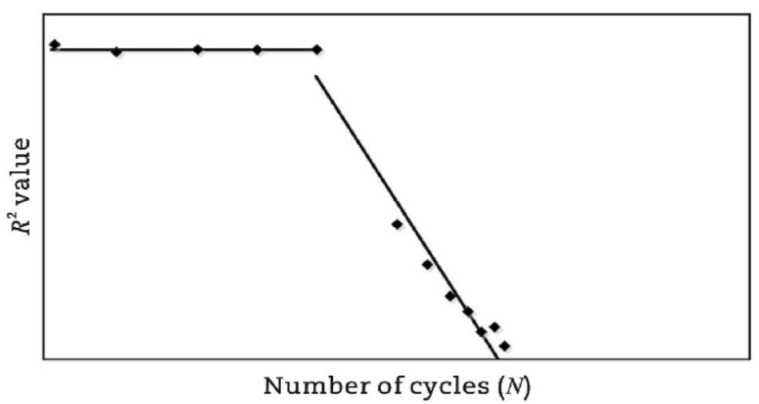
R-squared failure criterion [134].

Al-Khateeb and Shenoy [134] employed a statistical approach utilizing the coefficient of determination ($R^{2}$) to evaluate fatigue life by analyzing variations in stress–strain behavior. As the fatigue test progresses, damage will occur in the specimen, and the $R^{2}$ will decrease when using the input waveform function expression to fit the output test data, as shown in Figure 11. The criterion for fatigue failure is the first significant drop in the $R^{2}$ value, corresponding to the formation of irregular hysteresis loops. Nevertheless, this method remains subjective, as it depends on the manual detection of the point where distortion initiates or the $R^{2}$ value starts to decrease sharply.

To address the limitations of the $R^{2}$-based failure criterion, Kutay et al. [120] introduced a standard error parameter (se), computed using Equation (5), to quantify the degree of distortion throughout the entire testing duration. In the graph of the relationship between se and N, the point where se drastically decreases is considered the fatigue life.

(5) $$se=\sqrt{\frac{\sum_{i=1}^{n}{\left(Y_{f}-Y_{m}\right)}^{2}}{n-4}}\frac{100\%}{\left|Y_{f}^{*}\right|}$$

where $Y_{m}$ is the measured data, $Y_{f}$ is the fit data, n represents the total data points within a single cycle, and $Y_{f}^{*}$ represents the peak magnitude of the fitted sinusoidal wave.

(4) Specimen Homogeneity Criterion

When the uniaxial fatigue tests are performed in the laboratory, three axial extensometers measuring the axial strain at three locations are fixed around each specimen, which is depicted in Figure 5a. The measurement of axial strains and phase angles in three directions around the specimen, as well as their average values, can all be obtained through testing. Then, three relative axial strain amplitude differences and three phase angle differences can be calculated using Equations (6) and (7).

(6) $$∆\varepsilon_{axi}=\frac{\left(\varepsilon_{Aaxi}-\varepsilon_{Aax}\right)}{\varepsilon_{Aax}}\times 100\%$$

(7) $$∆\varphi_{i}=\varphi_{\varepsilon iax}-\varphi_{Aax}$$

where $∆\varepsilon_{axi}$ is the relative axial strain amplitude difference for the i direction, $\varepsilon_{Aaxi}$ is the average of the axial strain amplitude for the i direction, $\varepsilon_{Aax}$ is the average of the three axial strain amplitudes, $∆\varphi_{i}$ is the phase angle difference for the i direction, in degrees, $\varphi_{\varepsilon iax}$ is the phase angle corresponding to axial strain for the i direction, in degrees, and $\varphi_{Aax}$ is the average of the phase angle for the three axial directions, in degrees.

**Figure 12:**
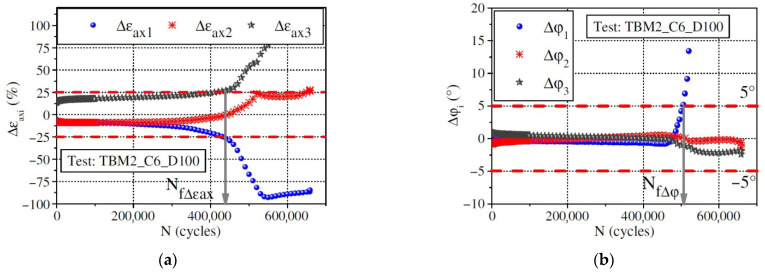
(**a**) Axial strain amplitude differences criterion; (**b**) phase angle axial displacement differences criterion [97].

The observed differences potentially reflect the homogeneity levels of both the strain and stress fields along the axial direction in specimens subjected to uniaxial cyclic loading. Thus, two criteria have been proposed by Ashayer Soltani [135] and Baaj [136], that is, the axial strain amplitude differences criterion ($N_{f∆\varepsilon ax}$) and the phase angle axial displacement differences criterion ($N_{f∆\varphi }$). The axial strain amplitude differences criterion, $N_{f∆\varepsilon ax}$, is the number of loading cycles required to achieve a 25% deviation for a given ${∆}_{\varepsilon axi}$. The phase angle axial displacement differences criterion, $N_{f∆\varphi }$, is the number of loading cycles requiring a $5^{{}^{\circ}}$ deviation for one ${∆}_{\varphi i}$. The definitions of the two criteria can be characterized by plotting the relationship between $∆\varepsilon_{axi}$, $∆\varphi_{i}$, and cycles (N), which is illustrated in Figure 12.

In summary, Table 2 systematically consolidates the conceptual frameworks and schematic representations of these fundamental failure criteria.

**Table 2:** Conceptual frameworks and schematic representations of the fundamental failure criteria.

Criteria	Indicator	Schematic Diagram	References
Stiffness modulus reduction criteria	Stiffness modulus	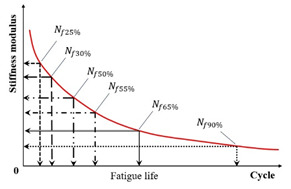	[28,31,111,112]
	Pseudo stiffness	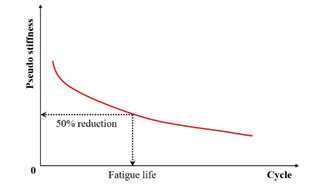	[114,115]
	Local modulus	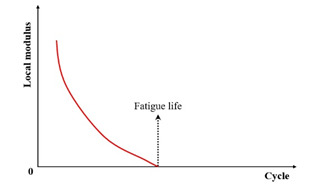	[123]
	Complete fracture	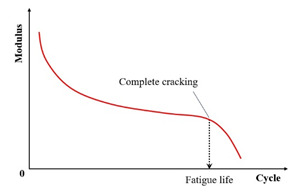	[31]
	Strain	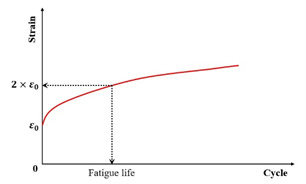	[98]
Phase angle criterion	Phase angle	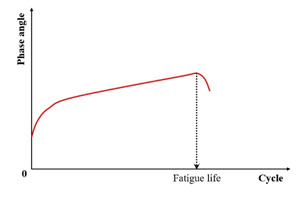	[19,31,132]
Fitting change point criterion	$R^{2}$ value/se value	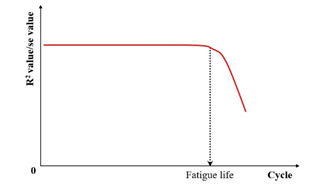	[134,137]
Specimen homogeneity criterion	Axial strain amplitude differences (${\Delta \varepsilon }_{axi}$ )	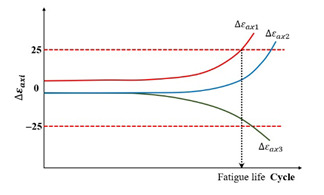	[135,136]
	Phase angle axial displacement differences (${\Delta \varphi }_{i}$ )	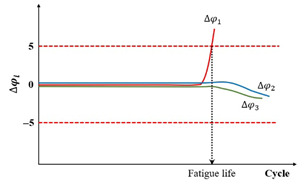	

#### 3.1.2. Failure Criteria of Energy-Based Models

**Figure 13:**
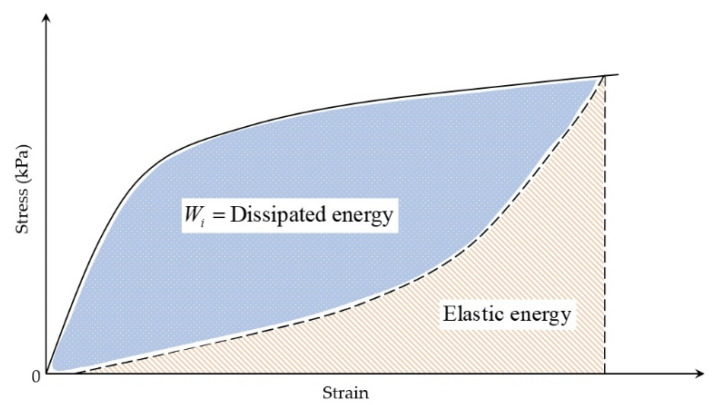
The schematic diagram of dissipated energy.

**Figure 14:**
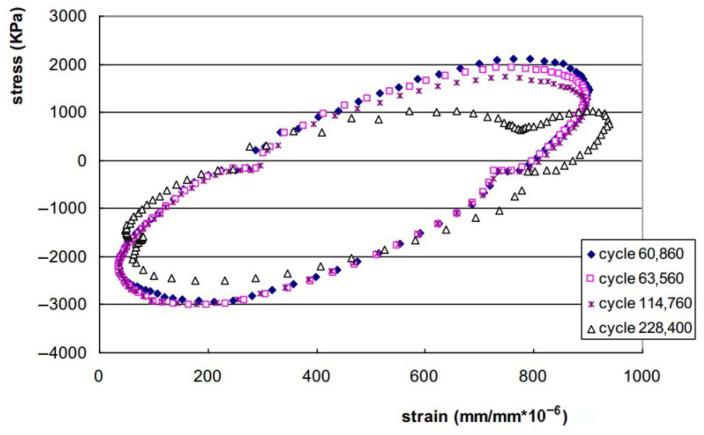
Different stress–strain hysteresis loops for the same mixture [138].

During fatigue testing involving cyclic loading and unloading, asphalt mixtures dissipate energy due to their inherent viscoelastic nature at typical service temperatures. This dissipated energy represents the portion consumed internally by the mixture through deformation mechanisms and microstructural damage under repeated stress. In contrast, elastic materials store all the input energy during loading (corresponding to the area beneath the load–deflection curve) and fully release it upon unloading. However, for viscoelastic materials, the path during unloading is different from that during loading, leading to hysteresis and dissipated energy, as indicated in Figure 13. This dissipated energy is quantified by the area within the stress–strain hysteresis loops, as illustrated in Figure 14.

The fundamental principles of dissipated energy in asphalt materials trace back to the seminal work of Chomton and Valayer [139] and Van Dijk et al. [140]. The dissipated energy can be calculated through Equation (8) [15,16,141,142,143].

(8) $$W_{i}=\pi \sigma_{i}\varepsilon_{i}\sin\delta_{i}=\pi \varepsilon_{i}^{2}{\left(S_{mix}\right)}_{i}\sin\delta_{i}$$

where $W_{i}$ is the dissipated energy in cycle i, J·m^−3^, $\delta_{i}$ is phase angle in cycle i, in degrees, ${\left(S_{mix}\right)}_{i}$ is the mix stiffness in cycle i, Pa, $\sigma_{i}$ is the stress in cycle i, Pa, and $\varepsilon_{i}$ is the strain amplitude in cycle i.

The amount of dissipated energy per loading cycle will change during the cyclic fatigue tests, as shown in Figure 14. Studies confirm that the dissipated energy per cycle escalates under controlled-stress conditions, but diminishes during controlled-strain testing [7,144]. The foundational correlation between the cumulative dissipated energy and mixture fatigue life was pioneered by Chomton and Valayer [139], as formalized in Equation (9). In addition, Van Dijk et al. [140] also reported a similar relationship with an exponent of 0.625 to that developed by Chomton and Valayer. However, these calculations do not take into account the changes in stiffness and the phase angle.

(9) $$W_{N_{f}}=\pi \sum_{i=0}^{N_{f}}\sigma_{i}\varepsilon_{i}\sin\delta_{i}=AN_{f}^{z}$$

where $W_{N_{f}}$ represents the total dissipated energy accumulated until failure, J·m^−3^, $N_{f}$ is the number of loading cycles to failure, and A and z are material constants determined experimentally.

(1) Energy Ratio Criteria

The energy ratio ($W_{n}$) approach, originally conceived by Van Dijk and Visser [144] and later refined by Pronk and Hopman [145,146] and Rowe [116], enables the graphical identification of dual fatigue stages via discontinuities in $W_{n}$ evolution versus cycle count N (Equation (10)).

(10) $$W_{n}=\frac{n\left(\pi \sigma_{0}\varepsilon_{0}\sin\delta_{0}\right)}{\pi \sigma_{i}\varepsilon_{i}\sin\delta_{i}}=\frac{nw_{0}}{w_{i}}$$

where $W_{n}$ is the energy ratio, n is the number of load cycles, σ is the stress, Pa, ε is the strain amplitude, $w_{0}$ and $w_{i}$ are the dissipated energy at start of the test and ith cycle, respectively, J·m^−3^, and δ is the phase angle, in degrees.

**Figure 15:**
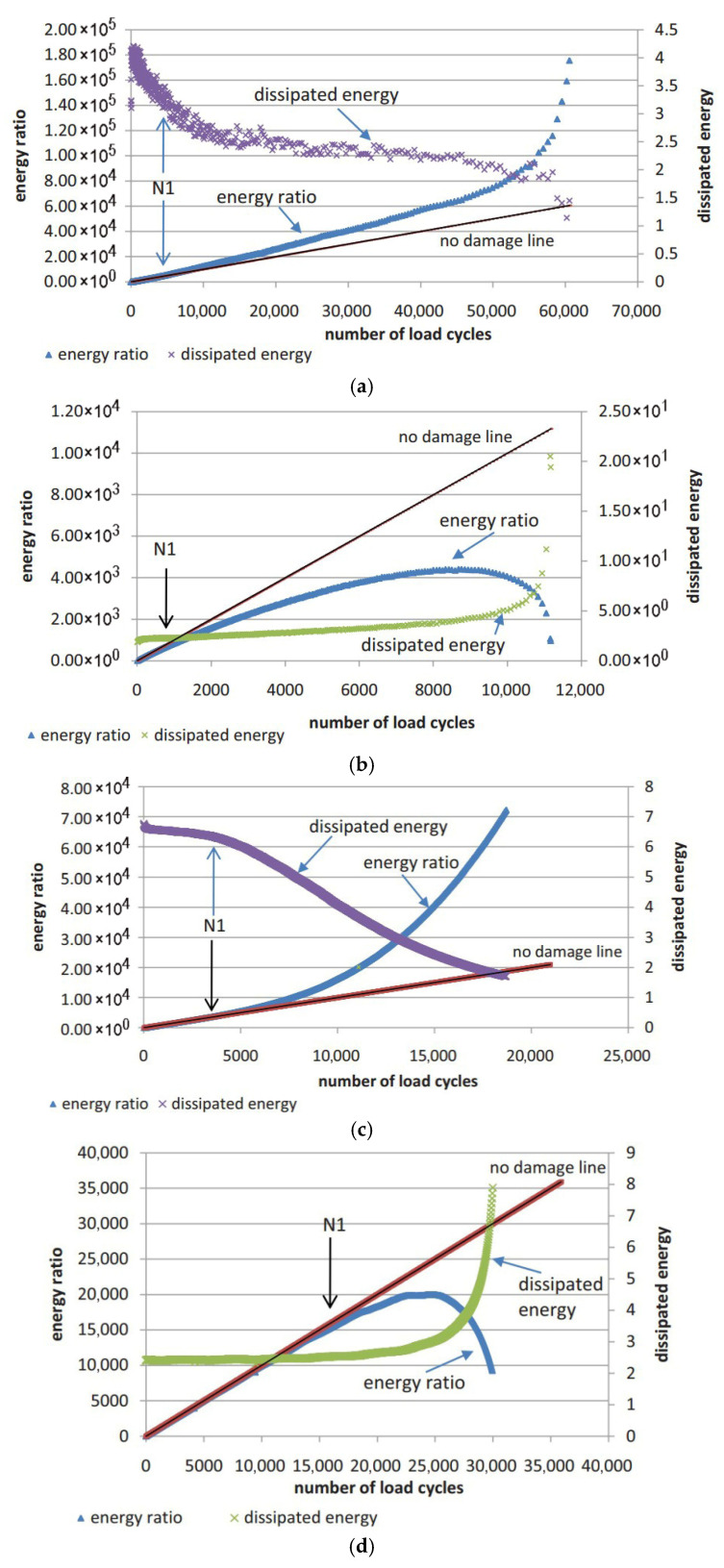
Energy ratio plot and dissipated energy plot for (**a**) controlled-strain mode for HMA mixture; (**b**) controlled-stress mode for HMA mixture; (**c**) controlled-strain mode for asphalt binder; and (**d**) controlled-stress mode for asphalt binder [128].

Specifically, the energy ratio for the controlled-strain and controlled-stress modes can be calculated using Equations (11) and (12), respectively. Shen and Lu [128] utilized the energy ratio versus loading cycles relationship, following the methodologies established by Hopmen et al. [146] and Rowe [116], to identify crack initiation sites and the critical point of microcrack-to-macro-crack progression in asphalt mixtures and binders subjected to controlled-strain or stress loading. Under controlled-strain loading, the first transition point (N1) on the dissipated energy–cycle curve is identified in Figure 15. A significant challenge in mathematically pinpointing the precise N1 value for both materials and loading modes arises because the energy ratio plot invariably shows progressive divergence from a straight line.

(11) $${For\;controlled\;strain\;mode:\;W}_{n}=\frac{n\left(\pi \sigma_{0}\varepsilon_{0}\sin\delta_{0}\right)}{\pi \sigma_{i}\varepsilon_{i}\sin\delta_{i}}=\frac{n\left(E_{0}^{*}\cdot \sin\delta_{0}\right)}{E_{i}^{*}\cdot \sin\delta_{i}}\approx \frac{nE_{0}^{*}}{E_{i}^{*}}$$

(12) $${For\;controlled\;stress\;mode:\;W}_{n}=\frac{n\left(\pi \sigma_{0}\varepsilon_{0}\sin\delta_{0}\right)}{\pi \sigma_{i}\varepsilon_{i}\sin\delta_{i}}=\frac{n\left(E_{i}^{*}\cdot \sin\delta_{0}\right)}{E_{0}^{*}\cdot \sin\delta_{i}}\approx \frac{nE_{i}^{*}}{E_{0}^{*}}$$

where $W_{n}$ is the energy ratio, n is the number of cycles, $\sigma_{0}$ and $\sigma_{i}$ are the stress at the initial (0) and ith cycle, respectively, Pa, $\varepsilon_{0}$ and $\varepsilon_{i}$ are the strain amplitude at the start of the test and the ith cycle, respectively, and $E_{0}^{*}$ and $E_{i}^{*}$ are the strain energy density at the start of the test and the ith cycle, respectively, J·m^−3^.

**Figure 16:**
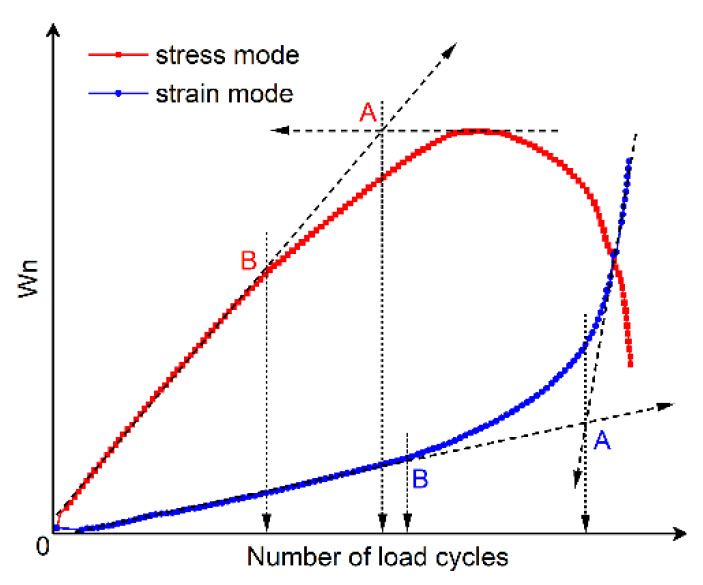
Energy ratio criteria of Pronk’s approach.

Furthermore, Pronk [147,148] established two fatigue life criteria for asphalt mixtures based on the energy ratio concept. Both criteria are discernible from the energy ratio versus loading cycles relationship, as illustrated in Figure 16.

Figure 16 shows two points, A and B, which are used to define the fatigue life based on the number of failure cycles. These points represent significant changes in the relationship, where point A is the intersection of the tangents between the two parts of the curve, and point B indicates the transition from the linear part to the nonlinear (curved) part. Although point A corresponds to an extended fatigue life compared to point B, the graphical method’s reliance on subjective interpretation introduces potential error. For controlled-strain tests, fatigue life is defined by point A, whereas point B determines the cycles to failure in controlled-stress tests.

As previously detailed for two points criteria (energy ratio criteria), the assessment of cycles to fatigue failure is significantly influenced by individual subjective conditions, the amount of test data, and the scale of the graph, etc. Therefore, it necessitates developing a consistent method or criterion that can accurately describe the process of damage of fatigue independently on the load modes and subjective effects.

(2) Stiffness Degradation Ratio Criterion

Rowe and Bouldin [121] analytically determined that the product *N × S* (where *N* = cycles, *S* = bending stiffness/complex modulus) locates the divergence of dissipated energy per cycle from linear behavior under controlled loading. The corresponding Equations are (13) and (14).

(13) $${\left(R_{\varepsilon }^{e}\right)}_{i}=\frac{N_{i}}{{\left(E^{*}\right)}_{i}}$$

(14) $${\left(R_{\sigma }^{e}\right)}_{i}=N_{i}\times {\left(E^{*}\right)}_{i}$$

where ${\left(R_{\varepsilon }^{e}\right)}_{i}$ and ${\left(R_{\sigma }^{e}\right)}_{i}$ are the stiffness reduction ratio at the ith cycle for strain-controlled and stress-controlled modes, respectively, ${\left(E^{*}\right)}_{i}$ is the stiffness (either a bending stiffness or complex modulus) at the ith cycle, Pa, and $N_{i}$ is the value at the ith cycle.

Rowe et al. [149] established that the point defined by Equations (13) and (14) aligned approximately with fatigue failure in conventional asphalt mixtures when assessed using the 50% stiffness reduction criterion. This approach, however, proved more effective for characterizing modified materials with lower stiffness, as these often undergo greater stiffness loss prior to the onset of sharp cracking. The methodology utilizing the stiffness degradation ratio ($N_{i}\times {\left(E^{*}\right)}_{i}$)—which yields a distinct peak facilitating unambiguous fatigue life determination—was subsequently incorporated into the ASTM standard for fatigue testing. This principle has also been adopted in the AASHTO T321 (2014) [150] protocol. The implementation of this ratio substantially mitigates the errors inherent in regression analysis, thereby enhancing the robustness of the testing standard [35,150].

Further research by Zeiada [151] refined the stiffness degradation ratio methodology initially proposed by Rowe and Bouldin [121]. This refinement involved normalizing the energy ratio through division by the material’s initial stiffness ($S_{0}$). The resulting enhanced stiffness degradation ratio is presented in Equation (15).

(15) $$Stiffness\;degradation\;ratio=\frac{N_{i}S_{i}}{S_{0}}$$

where $S_{0}$ is the initial stiffness measured at the 50th load cycle, Pa, $S_{i}$ is the stiffness measured at the ith cycle, Pa, and $N_{i}$ is the number at the ith cycle.

**Figure 17:**
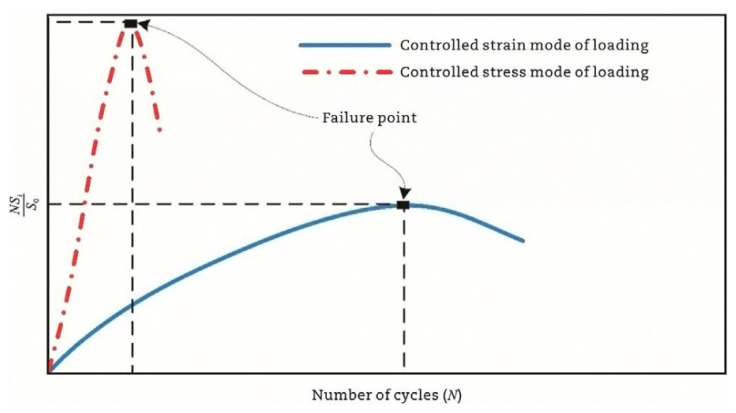
Stiffness degradation ratio for strain and stress mode of loading [7].

Figure 17 illustrates the relationship between the refined stiffness degradation ratio and the loading cycles for both the controlled-strain and controlled-stress modes. The peak value identified in this figure represents the material’s fatigue failure point. This graphical determination provides an unambiguous fatigue life criterion independent of subjective interpretation error.

Similarly, the AASHTO specification [152] defines the fatigue failure point as the load cycle corresponding to the peak value in the plot of stiffness multiplied by the load cycles versus load cycles. This peak directly indicates crack initiation within the specimen. Furthermore, ASTM specifications replace stiffness with the normalized modulus for fatigue failure determination. Specifically, the fatigue life is defined as the cycle count at which the normalized modulus × cycles product reaches its maximum value when plotted against loading cycles [35]. The governing equation for this parameter is defined as follows [121]:

(16) $$NM=\frac{S_{i}\times N_{i}}{S_{0}\times N_{0}}$$

where NM is the normalized modulus × cycles, $S_{i}$ is the flexural beam stiffness at cycle i, Pa, $N_{i}$ is the cycle i, $S_{0}$ denotes the initial flexural beam stiffness (Pa), typically measured at approximately 50 loading cycles, and $N_{0}$ represents the specific cycle count at which this initial stiffness determination occurs.

**Figure 18:**
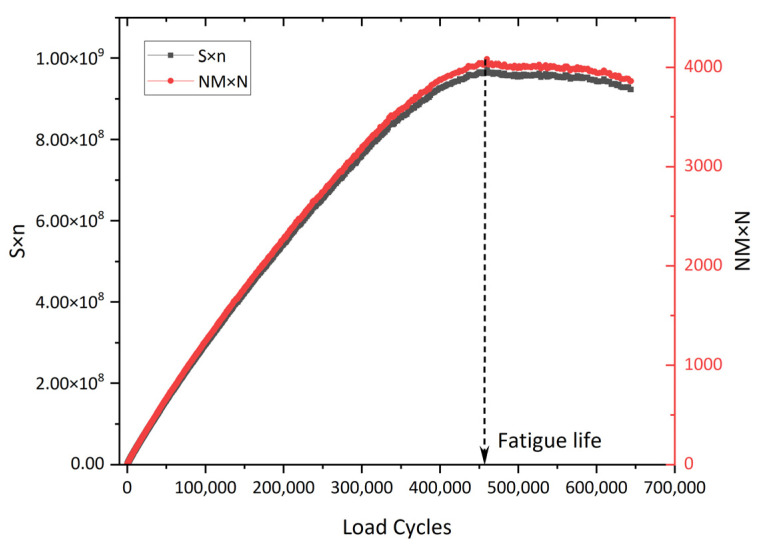
The failure criteria in AASHTO and ASTM.

The comparison of the failure criteria in AASHTO and ASTM is drawn in Figure 18 using the same set of fatigue test results. Thus, it can be seen that for the same set of fatigue test data, the same failure point can be obtained through the two failure criteria.

During fatigue testing at low strain amplitudes, the peak in the curve of the normalized modulus multiplied by cycles versus the cycle number occurs beyond the experimental duration. In such cases, the extrapolation failure point method is used to determine the fatigue life, which is shown in Equation (17) [153]. The failure point is estimated by solving the equation for the value of N where SR is equal to 0.500, that is, $Ln\left(-Ln\left(SR\right)\right)=-0.3665$, or 50% initial beam stiffness.

(17) $$Ln\left(-Ln\left(SR\right)\right)=\gamma \times Ln\left(N\right)+Ln\left(\lambda \right)$$

where $Ln\left(-Ln\left(SR\right)\right)$ is the natural logarithm of the additive inverse of the natural logarithm of SR, SR is the flexural beam stiffness ratio, $SR=\frac{S_{i}}{S_{0}}$, N is the number of cycles, γ is the slope of the linear regression of the $Ln\left(-Ln\left(SR\right)\right)$ versus $Ln\left(N\right)$, and $Ln\left(\lambda \right)$ is the intercept of the linear regression of the $Ln\left(-Ln\left(SR\right)\right)$ versus $Ln\left(N\right)$.

**Figure 19:**
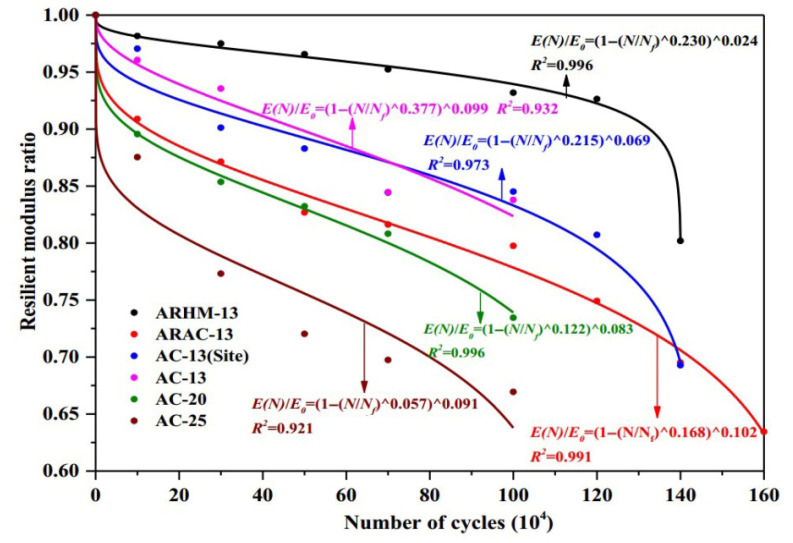
Resilient modulus (stiffness) ratio versus number of cycles [11].

Based on small-scale accelerated pavement testing data, Lv et al. [11] established a failure criterion for predicting the fatigue life of six asphalt mixtures. This criterion utilized the peak rate of stiffness reduction occurring during the transition from the quasi-stationary phase to the failure phase, as detailed in Figure 19. The stiffness reduction model proposed by Lv et al. is given in Equation (18). The accuracy and feasibility of using the maximum stiffness reduction rate as a failure criterion under different loading intervals have been verified.

(18) $$\frac{E\left(N\right)}{E_{0}}={\left[1-{\left(\frac{N}{k{\left(\frac{1}{\sigma }\right)}^{n}}\right)}^{\frac{1}{1-\alpha }}\right]}^{\frac{1}{1+\gamma }}$$

where $E\left(N\right)$ is the damaged resilient modulus in the Nth cycle, Pa, $E_{0}$ is the initial resilient modulus, Pa, N is the number of cycles, k and n are the fitting parameters, σ is the stress level, Pa, and α and γ are the material parameters, which are closely related to the stress amplitude and average stress, respectively.

**Figure 20:**
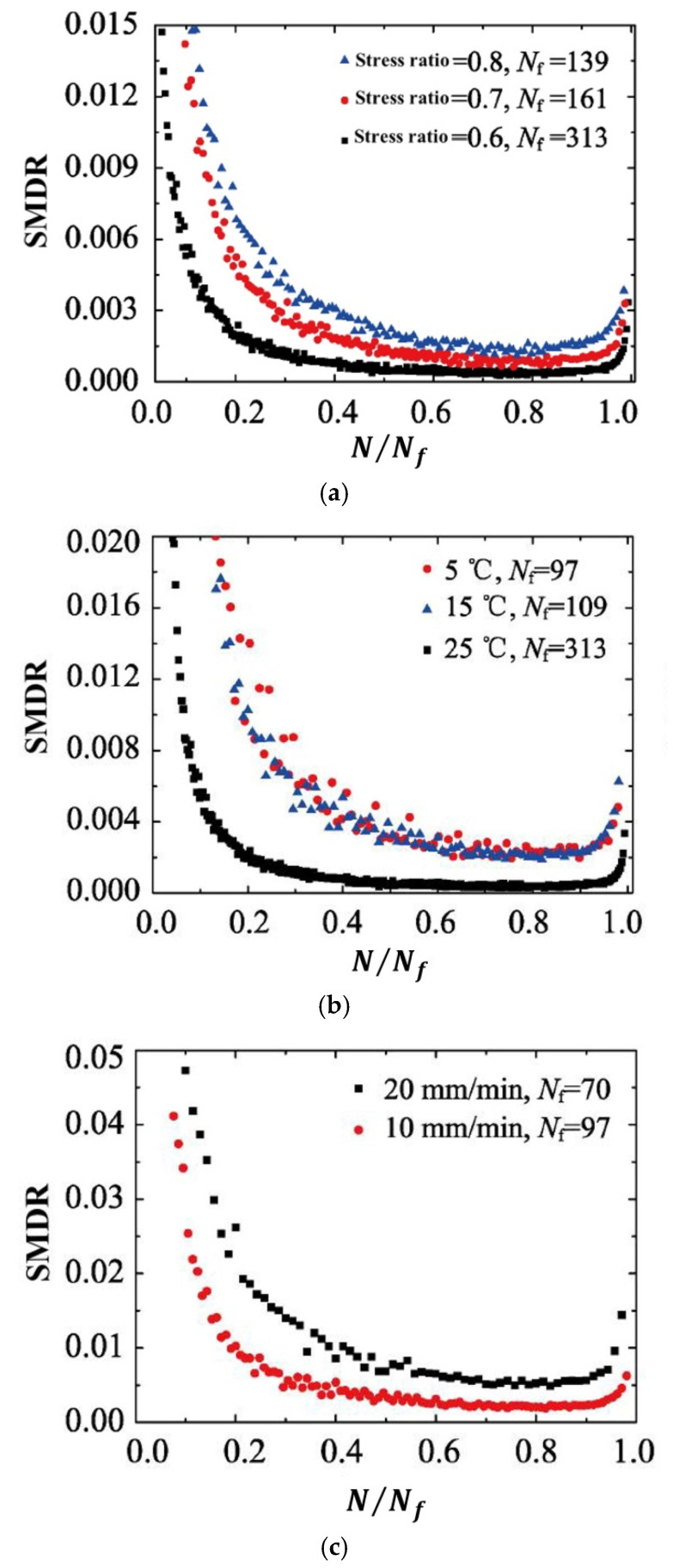
Stiffness modulus degradation ratio under different test conditions (modified based on Ref [154]): (**a**) under different stress ratios (25 °C, 10 mm/min); (**b**) under different temperatures (stress ratio = 0.6, 10 mm/min); and (**c**) under different load rates (stress ratio = 0.6, 5 °C).

Utilizing three-point flexural fatigue test data for rubber asphalt mixtures under controlled-stress conditions, Fang et al. [154] introduced the stiffness modulus degradation ratio (SMDR), as defined in Equation (19), to quantify the contribution of stiffness modulus degradation (SMD) to damage deformation. This parameter served as a metric for analyzing damage evolution and predicting fatigue life. Figure 20 illustrates SMDR variation across normalized loading cycles ($N/N_{f}$) under diverse testing conditions.

(19) $$SMDR=\frac{{SMD}_{N+1}-{SMD}_{N}}{{SMD}_{N}}$$

where the SMDR is the stiffness modulus degradation ratio, ${SMD}_{N}$ and ${SMD}_{N+1}$ are the stiffness modulus degradation at cycle N and N+1, respectively, MPa.

Although the SMDR parameter has been utilized by Fang et al. [154] for fatigue testing in the controlled-stress mode, this indicator can be extrapolated to controlled-strain fatigue testing. The fatigue failure point is defined as the cycle count corresponding to the transition between the secondary and tertiary damage stages. This approach might also serve as a potential criterion for fatigue failure.

(3) Stress Degradation Ratio Criterion

**Figure 21:**
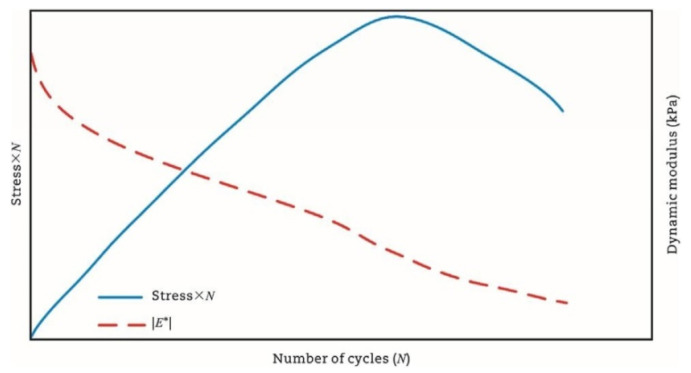
Stress degradation ratio [7].

**Figure 22:**
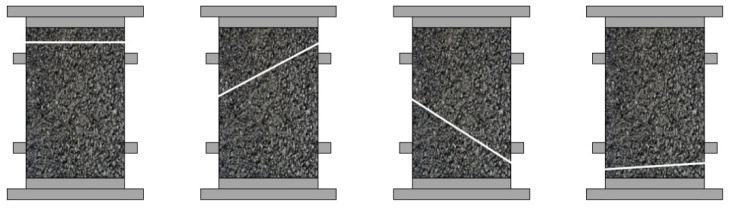
Schematic of end failure locations.

In further work, the stress degradation ratio criterion was proposed in a typical uniaxial fatigue test to eliminate the need for any on-specimen LVDT (linear variable differential transformer) measurements, resulting in defining the fatigue failure point accurately even in cases where the specimen fails outside the measurement points of the LVDT. Fatigue failure corresponds to the maximum value in the characteristic curve representing the product of stress amplitude and loading cycles versus the cycle count, as shown in Figure 21. Research has found that the stress degradation ratio criterion is the preferred criterion among various phenomenological models, as it is easy to measure, reduces the testing time, and is applicable to the end failure, in which the macro-crack develops outside the range of one or more axial deformation sensors, as illustrated in Figure 22.

(4) Dissipated Energy Ratio Criteria

The dissipated energy ratio (DER) constitutes a novel approach distinct from conventional energy ratio methods. Defined mathematically by Equation (20), the DER represents the ratio of the differential dissipated energy between consecutive loading cycles (i+1 and i) to the energy dissipated in cycle i. Bonnetti et al. [155] employed this concept to evaluate asphalt binder fatigue performance, establishing the failure point ($N_{p20}$) as the cycle count where the DER exhibits 20% deviation from the no-damage baseline. Equation (21) specifically determines $N_{p20}$ at this 20% deviation threshold.

(20) $$DER=\frac{W_{i+1}-W_{i}}{W_{i}}$$

where the DER is the dissipated energy ratio, and $W_{i+1}$ and $W_{i}$ are the dissipated energy in loading cycle i+1 and i, respectively, J·m^−3^.

(21) $$\%\;deviation=\frac{R-N}{N}\times 100$$

where R is the dissipated energy ratio, and N is the number of loading cycles.

Subsequent research introduced the ratio of the dissipated energy change (RDEC), formalized in Equation (22) as an advancement of the DER methodology. This refinement enables application to non-consecutive loading cycles, enhancing fatigue characterization in asphaltic materials. The RDEC quantifies the dissipated energy change between two arbitrary cycles relative to the initial cycle’s dissipated energy. The RDEC methodology and its predecessor (DER) were conceptualized by Ghuzlan and Carpenter [156], extending the foundational work by Carpenter and Jansen [157] and integrating the dissipated energy principles established in [116,145,146].

(22) $${RDEC}_{a}=\frac{{DE}_{a}-{DE}_{b}}{{DE}_{a}\left(b-a\right)}$$

where a and b are the loading cycle a and b, respectively, ${RDEC}_{a}$ is the averaged dissipated energy change ratio at cycle a relative to cycle b, and ${DE}_{a}$ and ${DE}_{b}$ are the dissipated energies at cycle a and b, respectively, kPa.

**Figure 23:**
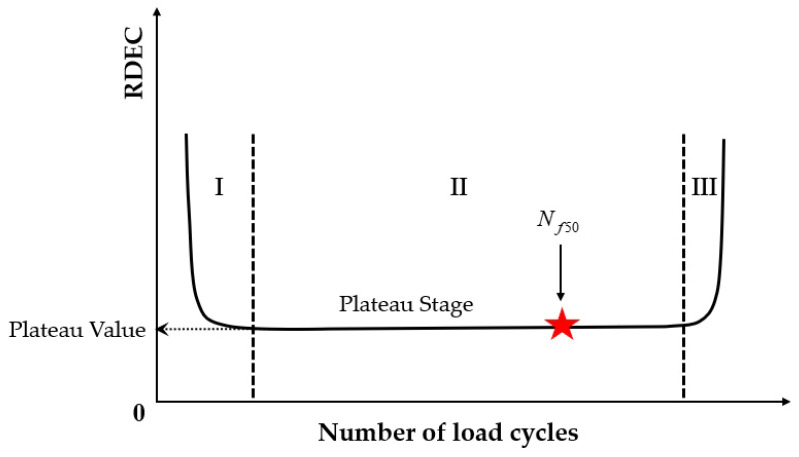
Typical RDEC plot with three behavior stages.

Figure 23 exhibits a characteristic three-stage RDEC evolution: Stage I demonstrates a rapid RDEC decline with cycling, Stage II sustains a near-constant plateau value (PV), and Stage III displays progressive RDEC growth. Carpenter and Shen [142] established the PV as a fundamental damage metric that intrinsically incorporates material properties and loading effects while excluding non-damaging energy components (e.g., thermal dissipation), thus exclusively quantifying damage-relevant energy dissipation in HMA. Compared to complex mechanical models, the RDEC approach preserves the conceptual simplicity of energy-based methodologies. Critically, controlled-strain testing reveals an inverse correlation between the PV magnitude and fatigue life for given HMA mixtures [16].

Ghuzlan and Carpenter [156] suggested that the cycle marking the Stage II–III transition (evidenced by an abrupt RDEC increase at the plateau terminus in Figure 23) coincides with unstable macroscopic cracking initiation. This critical transition defines the RDEC failure criterion. In some research, the failure criterion is considered more fundamental than the traditional 50% reduction in the stiffness criterion [15,16,158]. In addition, an improved dissipated energy failure criterion proposed by Daniel et al. [143] defined the point at which the dissipated energy ratio just exceeds the plateau value as the point failure.

**Figure 24:**
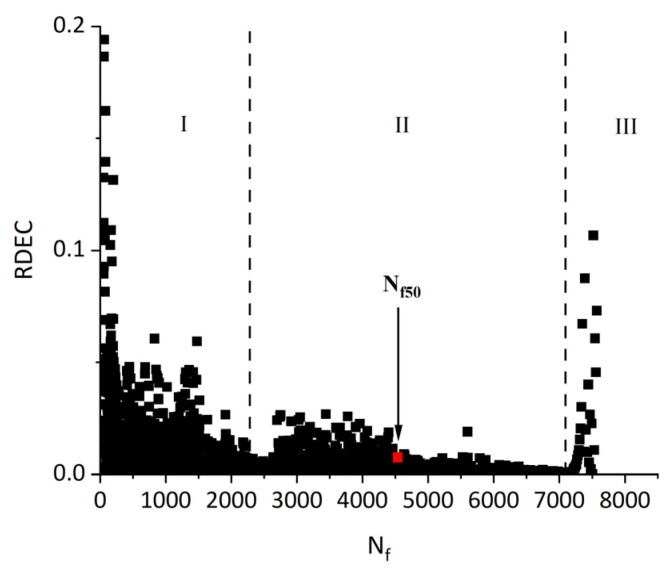
The relationship of the RDEC versus the number of load cycles and the indication of PV from fatigue testing.

However, due to the sharp fluctuations in fatigue data, it is difficult and complex to determine an accurate PV from the fatigue test data, as illustrated in Figure 24. Consequently, Carpenter and Shen [142] established the RDEC magnitude at a 50% stiffness reduction as the PV. This standardized approach leverages the consistent positioning of this point within the plateau stage, thereby mitigating the random errors inherent in data processing methodologies. Shen and Carpenter [16] further established that the relationship between PV and $N_{f50}$ is fundamental and persists irrespective of the loading levels (including normal and low damage), loading modes (stress- or strain-controlled), mixture types, or testing parameters (e.g., frequency, rest periods). The equation of PV-$N_{f50}$ is shown as Equation (23). Although Bhasin et al. [159] concluded that the RDEC approach is contingent upon the loading mode, as evidenced by the fatigue test results of the fine aggregate matrix, the distinctive relationship of PV-$N_{f50}$ and the analogous equation have been validated as reasonable by numerous scholars [160,161,162].

(23) $$PV=0.4428\times {N_{f50}}^{-1.1102}$$

**Figure 25:**
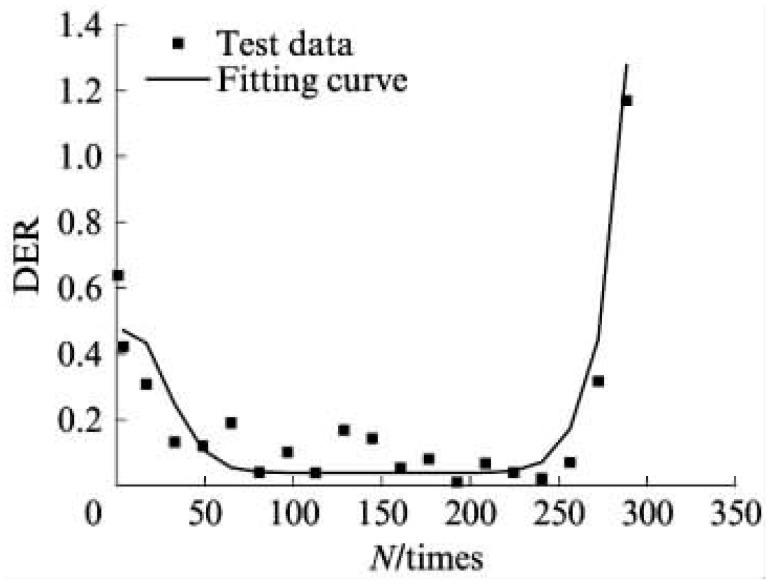
Contrast result of DER (the former version of RDEC) of rubber asphalt mixture [163].

In order to determine the plateau value of the RDEC in the second stage, Sun et al. [163] proposed an equation, as shown in Equation (24), to fit the relationship between the dissipated energy in adjacent loading cycles and the number of fatigue cycles in rubber asphalt mixtures using the fatigue testing data obtained from three-point flexural fatigue tests under the stress-controlled mode. The curve of the RDEC versus the number of loading cycles is depicted in Figure 25. This method may be a promising approach to accurately determine the significant change point location from the second stage to the third stage.

(24) $$RDEC=\frac{a_{2}\left[\left|{\left(N+1-a_{3}\right)}^{a_{4}}\right|-\left|{\left(N-a_{3}\right)}^{a_{4}}\right|\right]}{a_{1}+a_{2}\left|{\left(N-a_{3}\right)}^{a_{4}}\right|}$$

where the RDEC is the ratio of the dissipated energy change, N is the number of cycles, and $a_{1}$, $a_{2}$, $a_{3}$, and $a_{4}$ are the fitting parameters.

Furthermore, research shows that the RDEC appears in a dispersed state with significant fluctuations in the second stage, indicating substantial variability in the RDEC within adjacent cycles that characterize the damage of asphalt mixtures. This finding indicates that the RDEC inadequately characterizes the material’s nonlinear damage evolution across varying conditions [164]. Consequently, Fang et al. [165] proposed the ratio of the cumulative dissipated energy change (RCDEC), defined using monotonically increasing cumulative dissipated energy to better characterize continuous nonlinear damage progression in asphalt mixtures under diverse loading scenarios. The RCDEC is calculated using Equations (25) and (26).

(25) $$R_{CDEC}=\frac{C_{DE,N+1}-C_{DE,N}}{C_{DE,N}}$$

(26) $$C_{DE,N}=\sum_{k=1}^{N}D_{E,k}$$

where $C_{DE,N}$ and $C_{DE,N+1}$ are the cumulative dissipated energy at the number of N and N+1 cycles, respectively, J·m^−3^, $D_{E,k}$ is the dissipated energy at the kth cycle, J·m^−3^, and $R_{CDEC}$ is the ratio of the cumulative dissipated energy change.

**Figure 26:**
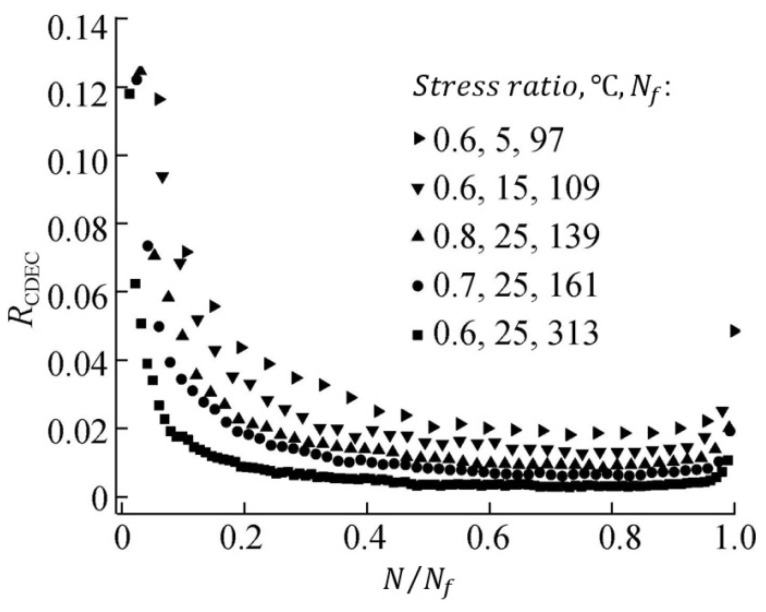
The relationship of $R_{CDEC}$ versus $N/N_{f}$ (modified based on Ref [165]).

The fatigue resistance of asphalt mixtures can be effectively characterized using the RCDEC. Plotting the RCDEC against $N/N_{f}$ under various stress ratios and temperatures during three-point flexural fatigue testing (Figure 26) enables this distinction. For stress-controlled tests, lower RCDEC PVs correlate with a longer fatigue life in HMA mixtures, aligning with Shen and Carpenter’s findings [16]. Consequently, the load cycle count, triggering a rapid RCDEC increase at the Stage II-III transition, can serve as a fatigue failure criterion, offering a potential method for predicting asphalt mixtures’ fatigue life.

(5) Fracture Energy Criteria

Zhang et al. [166] and Roque et al. [167] introduced the damage threshold concept, observing that micro-damage in asphalt mixtures exhibits an autonomous healing capacity, while macro-damage does not. This signifies that sub-threshold damage is fully healable, whereas damage exceeding this threshold becomes irrecoverable. Consequently, the threshold delineates macro-crack development (macro-damage) throughout crack initiation and propagation, irrespective of timing or location within the mixture.

**Figure 27:**
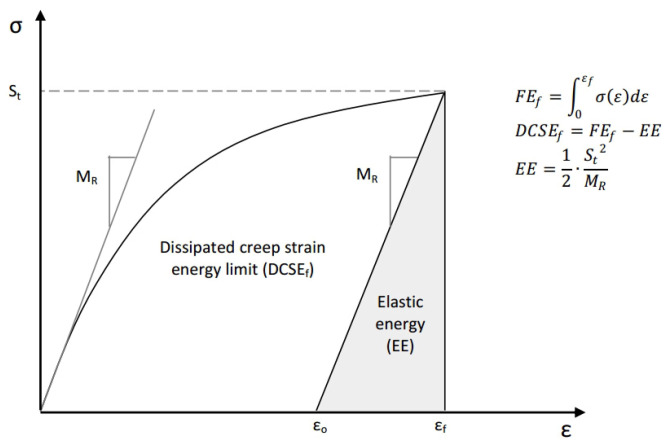
Determination of failure limits in asphalt mixtures.

To quantify the failure limits for specific fracture modes (repeated or single loading), the energy thresholds of the dissipated creep strain energy (DCSE) and fracture energy (FE) were introduced [168]. As illustrated in Figure 27, tensile strength testing provides these critical values, enabling failure assessment in asphalt mixtures.

The fracture energy limit (${FE}_{f}$) corresponds to the stress–strain curve’s integrated area, while the dissipated creep strain energy limit (${DCSE}_{f}$) equals ${FE}_{f}$, minus the elastic energy (EE) at fracture. The EE is defined using the resilient modulus ($M_{R}$) and tensile strength ($S_{t}$). Crucially, both the ${DCSE}_{f}$ and ${FE}_{f}$ represent the inherent material properties of asphalt mixtures, independent of the loading mode, rate, and specimen geometry [169].

**Figure 28:**
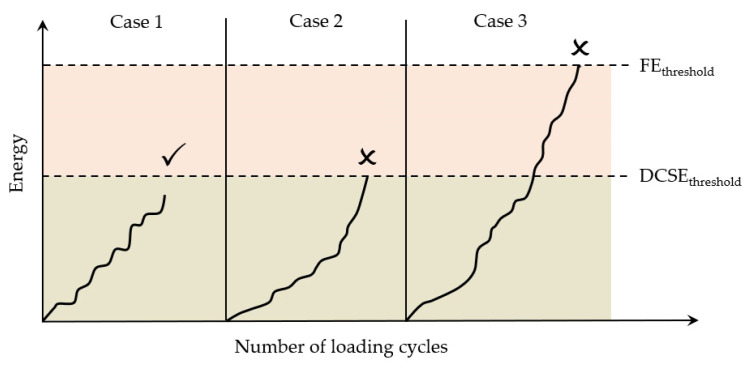
Diagram of damage threshold.

**Figure 29:**
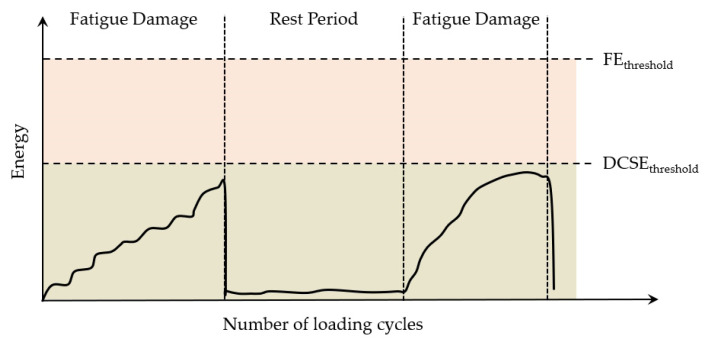
Schematic diagram of healing effect of asphalt mixture.

Under the repeated load cyclic fatigue, fatigue cracks will develop when either of these two thresholds is exceeded, which is shown in Figure 28. In actual pavements, due to the healing effect of asphalt mixtures, the accumulated energy in the asphalt mixture may never reach this critical standard, as shown in Figure 29; hence, cracks will not propagate further in asphalt concrete pavements. When the accumulated energy reaches the dissipated creep strain energy (DCSE) threshold for macroscopic damage to the asphalt mixture, cracks begin to form in the asphalt mixture.

In summary, Table 3 systematically consolidates the conceptual frameworks and schematic representations of these energy-based failure criteria.

### 3.2. Mechanistic Approaches

Empirical models inadequately explain the mechanisms of fatigue damage initiation in asphalt mixtures and the resulting material/structural characteristics. Consequently, researchers are increasingly applying mechanical theories to analyze asphalt mixture fatigue damage behavior, aiming for more accurate fatigue life predictions [13,72,170,171]. In the mechanical approach, fracture mechanics and continuum damage mechanics are the two main methods used to predict pavement performance, differing in that fracture mechanics assumes that microcracks or defects within the material are inherently present, focusing primarily on the mechanism of crack propagation without considering the crack initiation process, while continuum damage mechanics assumes that damage stems from microcracks distributed throughout the entire continuum. In essence, mechanical models provide a more fundamental analysis of fatigue damage than empirical methods.

#### 3.2.1. Fracture Mechanics Models

**Figure 30:**
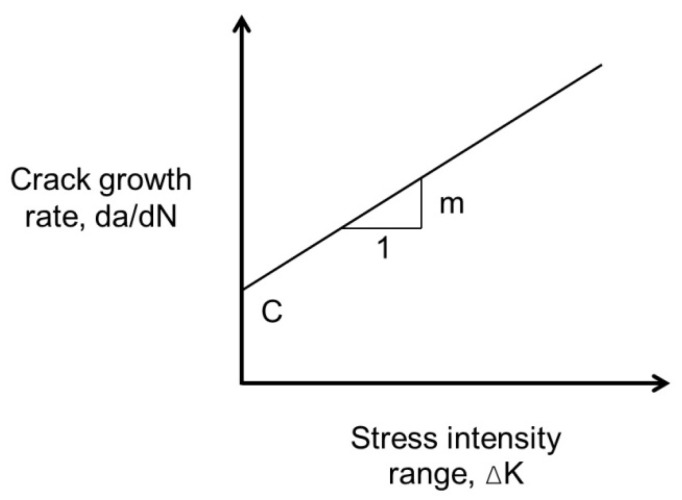
The diagram of Paris’s Law.

The fracture mechanics approach characterizes material fatigue failure by the cycle count necessary for a crack to propagate from an initial size to a critical threshold. Paris and Erdogan [172] experimentally observed in the early 1960s that log–log plots of the crack growth rate against stress intensity factor ranges yielded linear relationships, establishing the principle now termed Paris’ Law. The equation is as shown in Equation (27), and the figure is as depicted in Figure 30.

(27) $$\frac{da}{dN}=C{\left(∆K\right)}^{m}$$

where $\frac{da}{dN}$ is the crack growth rate, a is the crack length, C and m are material constants, K is the stress intensity factor, $∆K=K_{max}-K_{min}$, which is the stress intensity factor range, and N is the number of applied loading cycles.

**Figure 31:**
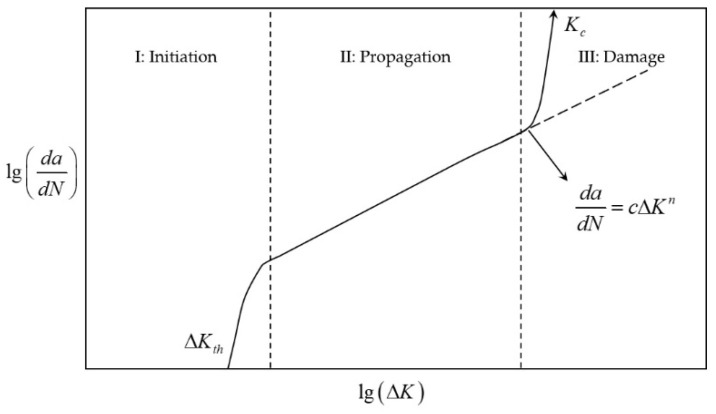
Rate of crack growth versus stress intensity factor in the three phases of crack propagation.

As conceptualized by Erkens and Moraal [173] (Figure 31), the fatigue cracking mechanism evolves through three sequential phases.

The initiation phase commences with micro-scale plastic deformation at the crack tip, confined within the grain dimensions. This stage exhibits material stiffness reduction driven by micro-crack nucleation, a process appropriately modeled by damage mechanics principles.

Subsequently, the propagation phase emerges through coalescence of micro-cracks into dominant macro-cracks. Here, linear elastic fracture mechanics provides a valid analytical framework due to limited plasticity.

The process culminates in the damage phase, where elastic fracture mechanics analysis becomes invalidated by extensive plastic zones. Unrestricted crack advancement precipitates accelerated material degradation, culminating in structural failure.

(1) J-integral Approach

The fatigue-induced energy dissipation in asphalt mixtures encompasses both elastic and viscous components. The rate of change in the dissipated energy, which indicates the onset and progression of damage or cracks in asphalt mixtures, is primarily determined by their viscous component. Crucially, the J-integral quantifies this energy change rate per unit crack area extension and demonstrates a strong loading time dependence in viscoelastic materials, as shown in Equation (28) [174].

(28) $$J=\frac{dW}{bdc}=\frac{dW/dN}{bdc/dN}$$

where W is the dissipated energy, J·m^−3^, b is the width of the specimen, m, dc is the change in the crack length, m, bdc is the change in the crack area, m^2^, dW is the corresponding change in the dissipated energy, J·m^−3^, and dW/bdc is the rate of change in the dissipated energy with respect to the change in the crack area.

Under plane strain conditions for linear elastic materials, the Paris–Erdogan law of fracture mechanics provides the theoretical foundation linking the J-integral to the stress intensity factor (SIF), K, as expressed in Equation (29). This relationship enables the correlation of the crack growth rate with the J-integral. Combining Paris’ law with the J-integral yields Equation (30). When applied specifically to linear elastic material behavior, the J-integral formulation of Paris’ law simplifies so that the material constants $A^{\prime }$ and $n^{\prime }$ are equivalent to A and n, respectively (Equations (31) and (32)).

(29) $$J=\frac{K^{2}}{E}\left(1-v^{2}\right)$$

(30) $$\frac{da}{dN}=A^{\prime }J^{n^{\prime }}$$

where K is the stress intensity factor, E is the Young’s modulus, Pa, v is the Poisson’s ratio, and $A^{\prime }$ and $n^{\prime }$ are the material parameters.

(31) $$n^{\prime }=\frac{n}{2}$$

(32) $$A^{\prime }=A{\left(\frac{E}{1-v^{2}}\right)}^{n}$$

Experimentally, the crack growth rates are commonly determined by tracking the crack surface via digital image correlation (DIC). Concurrently, J-integral values corresponding to these crack lengths are computed using finite element (FE) models. The J-integral methodology readily incorporates the viscoelastic correspondence principle for simulating fatigue crack growth.

(2) Pseudo J-integral Approach

The J-integral formulation of Paris’ law remains applicable for analyzing crack propagation in nonlinear viscoelastic materials. Although the parameters K (Equation (27)) and J (Equation (27)) originate from linear elastic and elastic–plastic fracture mechanics frameworks, both methodologies inherently retain time-dependent components within their respective measurements of stress, strain, and dissipated energy. Schapery [175] leveraged the elastic–viscoelastic correspondence principle to isolate viscoelastic contributions during crack growth modeling. This principle enables the use of a pseudo J-integral ($J_{R}$) form of Paris’ law (Equation (33)), defined as the dissipated pseudo strain energy rate per unit crack surface area. For viscoelastic materials like HMA, $J_{R}$ provides enhanced accuracy in predicting micro-crack growth by explicitly addressing time-dependent behavior.

(33) $$J_{R}=\frac{\partial W}{\partial s}=\frac{\partial W}{\partial N}\frac{\partial N}{\partial s}=\frac{\partial W/\partial N}{\partial s/\partial N}$$

The pseudo J-integral form ($J_{R}$) of Paris’ law, as described in Equation (34), can be utilized to predict the propagation of the micro-cracks.

(34) $$\frac{da}{dN}=A^{\prime }{J_{R}}^{n^{\prime }}$$

Solving Equation (34) for $N_{i}$ gives Equation (35).

(35) $$N_{i}=\frac{\frac{n^{\prime }+1}{2n^{\prime }+1}{\bar{C}}_{i}^{\frac{{2n}^{\prime }+1}{n^{\prime }+1}}}{{A^{\prime }}^{\frac{1}{n^{\prime }+1}}{\left(\frac{b}{4\pi }\right)}^{\frac{n^{\prime }}{n^{\prime }+1}}}$$

Consequently, accurately determining the dissipated pseudo strain energy (DPSE) during fatigue testing is critical. This measurement directly enhances the accuracy and applicability of the J-integral for characterizing crack growth in hot mix asphalt (HMA) materials.

#### 3.2.2. Viscoelastic Continuum Damage (VECD) Model

The viscoelastic continuum damage (VECD) model and its simplified closed-form variant (S-VECD) are derived from Schapery’s foundational research on viscoelastic fracture and distributed damage [176,177,178]. The VECD framework comprises four core components: (1) the pseudo strain ($\varepsilon^{R}$) function, incorporating linear viscoelasticity and time–temperature superposition (Equation (36)); (2) the pseudo strain energy density ($W^{R}$) function (Equation (37)); (3) the constitutive stress (σ) versus pseudo strain ($\varepsilon^{R}$) relationship (Equation (38)); and (4) the governing damage evolution law (Equation (39)).

(36) $$\varepsilon^{R}=\frac{1}{E_{R}}\int_{0}^{t}E\left(\xi -\tau \right)\frac{d\varepsilon }{d\tau }d\tau$$

(37) $$W^{R}=\frac{1}{2}{\left(\varepsilon^{R}\right)}^{2}C$$

(38) $$\sigma =\frac{dW^{R}}{d\varepsilon^{R}}=C\times \varepsilon^{R}$$

(39) $$\frac{dS}{d\xi }={\left(-\frac{\partial W^{R}}{\partial S}\right)}^{\alpha }$$

where $E\left(\xi \right)$ is the linear viscoelastic relaxation modulus, Pa, τ is the integration term, ξ is the reduced time, $E_{R}$ is the reference modulus (taken as 1), Pa, C is the pseudo secant stiffness (material integrity), N/m, and S is the damage.

Central to the VECD framework is the damage characteristic curve, defining the relationship between the damage magnitude (S) and the pseudo secant modulus (C), representing material integrity [179]. Daniel and Kim [180] established the VECD model as a foundational element for a streamlined fatigue testing protocol. Critically, research demonstrates that the material damage characteristics—independent of loading conditions and consistent with the time–temperature superposition principle routinely applied to low-strain dynamic modulus analysis—remain valid under elevated damage states [180,181]. This capability dramatically streamlines the required experimental procedures. Subsequently, Underwood et al. [182] developed a simplified damage model variant (S-VECD) specifically adapted for cyclic direct tension test data on asphalt mixtures, building upon the contributions of Daniel and Kim [180] and Chehab et al. [181].

The viscoelastic continuum damage (VECD) model finds broad application in predicting the fatigue performance of asphalt mixtures and pavements across varying conditions, based on limited experimental data [182,183]. In addition, the VECD or S-VECD modeling approach has been extended to asphalt binders tested using torsional loading in a dynamic shear rheometer (DSR) [21,127,184,185,186,187,188]. Hence, fatigue resistance parameters from the Linear Amplitude Sweep (LAS) test are combined with the S-VECD framework, facilitating fatigue life prediction at user-specified strain amplitudes and temperatures via limited experimental inputs.

While mechanistic approaches such as the VECD and S-VECD models have been proposed to quantify the dissipated energy, the models cannot predict the fatigue failure automatically without the criteria. Consequently, establishing robust failure criteria is crucial within the VECD (or S-VECD) framework. These criteria delineate the valid domain of the continuum damage model and enable the consistent prediction of material fracture corresponding to macro-crack formation.

(1) Pseudo Stiffness Criterion

In early research, the value of the pseudo stiffness was directly used to indicate the fatigue failure for the VECD model. Typically, pseudo stiffness values of 0.5 or 0.25 are used in the work of researchers [137,180,181,189,190]. Furthermore, Hou et al. [191] observed that a pseudo stiffness value near 0.5 aligned with the experimental results at 5 °C, whereas values approaching 0.25 better represented the data at approximately 19 °C and higher temperatures.

The failure criterion for the S-VECD model, known as the pseudo stiffness at failure (i.e., the C-value at the phase angle drop), was introduced by Hou et al. [191]. Based on experimental data from 12 mixtures tested across different temperatures, they postulated that failure initiates when the pseudo stiffness (C) attains a critical value ($C_{f}$). However, the main issue with the criterion is the high variability observed by researchers in the experimental data. Therefore, the critical pseudo stiffness parameter fails to serve as a robust criterion for failure prediction.

(2) $G_{0}^{R}$-based Criterion

**Figure 32:**
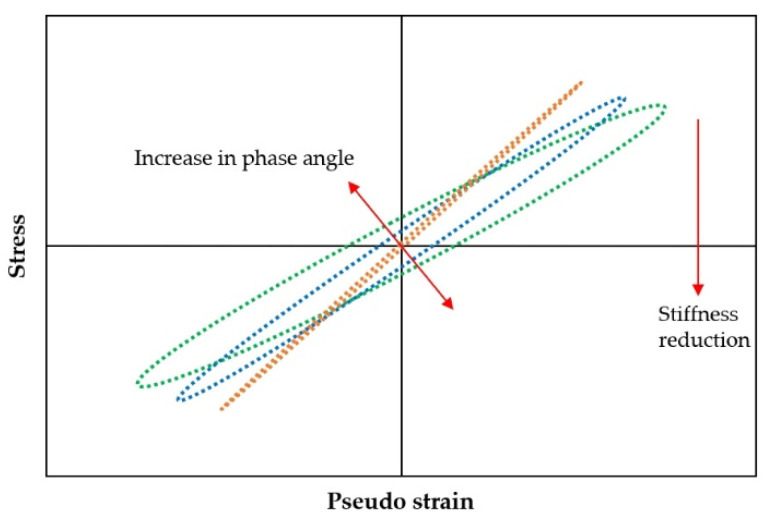
Pseudo hysteresis loops for controlled crosshead (CX) cyclic direct tension tests.

**Figure 33:**
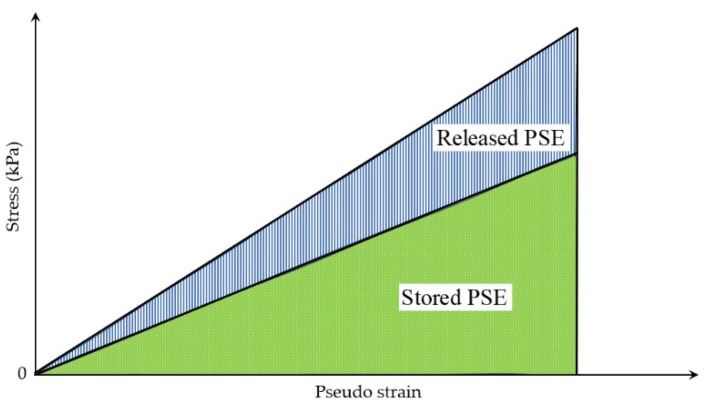
Pseudo strain energy compositions.

Within the VECD modeling framework, Zhang et al. [126] introduced a dissipated pseudo energy criterion. This criterion relies on the concept of released pseudo strain energy, defined as the difference between the current stored maximum pseudo strain energy and its corresponding undamaged state, attributed solely to stiffness reduction. This approach yields consistent and accurate fatigue failure predictions, corresponding to the experimentally observed phase angle drop. The pseudo strain energy (PSE), sometimes termed the dissipated pseudo strain energy (DPSE) in the literature, quantifies the energy dissipation exclusively associated with crack development and permanent deformation, excluding all viscoelastic effects. It is computed by replacing the actual strain with the equivalent pseudo strain. Represented as the hysteresis loop in stress–pseudo strain space (Figure 32), the PSE is determined by integrating the area under the stress versus the pseudo strain curve during cyclic fatigue tests, using Equations (40) and (41) [126] (Figure 33).

(40) $$W_{i}^{R}=\pi \sigma_{i}\varepsilon_{i}^{R}\sin\left(\varphi_{i}-\varphi \right)$$

(41) $$\varepsilon_{i}^{R}=E^{*}\varepsilon_{i}\sin\left(\omega t+\varphi \right)$$

where $W_{i}^{R}$ is the dissipated pseudo strain energy, $\varepsilon_{i}^{R}$ is the pseudo strain amplitude at cycle i, $\sigma_{i}$, $\varepsilon_{i}$, and $\varphi_{i}$ are the stress amplitude, strain amplitude, and phase angle measured at cycle i, respectively, $E^{*}$ is the complex modulus of the undamaged material at the specified reduced frequency, Pa, and φ is the phase angle, in degrees.

Figure 33 illustrates that the pseudo strain energy comprises two distinct components: the total released pseudo strain energy ($W_{C}^{R}$) and the total stored pseudo strain energy ($W_{S}^{R}$). Equations (42)–(44) and (45)–(47) are used to calculate these energy components under controlled-strain and stress loading modes, respectively [126,192,193].

(42) $${\left(W_{S,max}^{R}\right)}_{i}=\frac{1}{2}{\left(\sigma_{0,ta}\right)}_{i}{\left(\varepsilon_{0,ta}^{R}\right)}_{i}=\frac{1}{2}{\left(\varepsilon_{0,ta}^{R}\right)}_{i}^{2}$$

(43) $${\left(W_{S}^{R}\right)}_{i}=\frac{1}{2}{\left(\sigma_{0,ta}\right)}_{i}{\left(\varepsilon_{0,ta}^{R}\right)}_{i}=\frac{1}{2}{\left(C(S)\right)}_{i}{\left(\varepsilon_{0,ta}^{R}\right)}_{i}^{2}$$

(44) $${\left(W_{C}^{R}\right)}_{i}={\left(W_{S,max}^{R}\right)}_{i}-{\left(W_{S}^{R}\right)}_{i}=\frac{1}{2}\left(1-{\left(C(S)\right)}_{i}\right){\left(\varepsilon_{0,ta}^{R}\right)}_{i}^{2}$$

(45) $${\left(W_{S,max}^{R}\right)}_{i}=\frac{1}{2}{\left(\sigma_{0,ta}\right)}_{i}{\left(\varepsilon_{0,ta}^{R}\right)}_{i}=\frac{1}{2}{\left(\sigma_{0,ta}\right)}_{i}^{2}$$

(46) $${\left(W_{S}^{R}\right)}_{i}=\frac{1}{2}{\left(\sigma_{0,ta}\right)}_{i}{\left(\varepsilon_{0,ta}^{R}\right)}_{i}=\frac{1}{2}\frac{1}{{\left(C(S)\right)}_{i}}{\left(\sigma_{0,ta}\right)}_{i}^{2}$$

(47) $${\left(W_{C}^{R}\right)}_{i}={\left(W_{S,max}^{R}\right)}_{i}-{\left(W_{S}^{R}\right)}_{i}=\frac{1}{2}\left(1-\frac{1}{{\left(C(S)\right)}_{i}}\right){\left(\sigma_{0,ta}\right)}_{i}^{2}$$

where ${\left(W_{S,max}^{R}\right)}_{i}$ is the maximum stored pseudo strain energy at cycle i, J, ${\left(W_{S}^{R}\right)}_{i}$ is the stored pseudo strain energy at cycle i, J, ${\left(W_{C}^{R}\right)}_{i}$ is the total released pseudo strain energy at cycle i, J, ${\left(\sigma_{0,ta}\right)}_{i}$ and ${\left(\varepsilon_{0,ta}^{R}\right)}_{i}$ are the peak to peak stress and pseudo strain at cycle i respectively, and ${\left(C(S)\right)}_{i}$ is the magnitude-based pseudo stiffness at cycle i, Pa.

**Figure 34:**
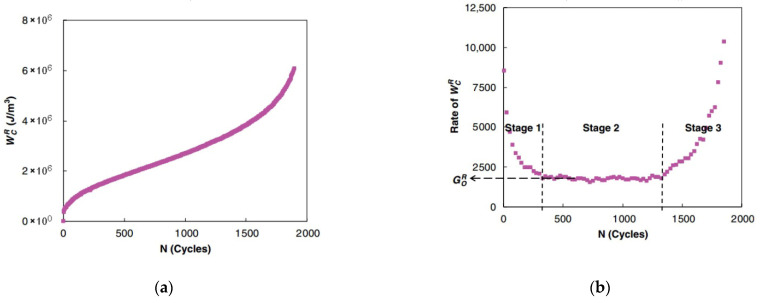
Zhang et al.’s approach [133]: (**a**) the history of $W_{C}^{R}$; (**b**) the rate of $W_{C}^{R}$.

Under controlled-strain loading, $W_{C}^{R}$ (total released pseudo strain energy) depends on the pseudo strain amplitude ($\varepsilon_{0,ta}^{R}$) and pseudo stiffness C(S). This key energy metric quantifies the total dissipated energy originating from both the external loading and the material itself. Zhang et al. [126] found that the relationship between $W_{C}^{R}$ and the number of cycles for all CX tests could be divided into three stages, which is depicted in Figure 34.

During the initial loading cycles, $W_{C}^{R}$ declines rapidly. This behavior corresponds to the evolving stress–strain response of the specimen during CX cyclic testing. Subsequently, the whole trend of the rate of $W_{C}^{R}$ maintains a constant value within certain limits in Stage 2, as illustrated in Figure 34b. The $G_{0}^{R}$ criterion, defined as the rate of the pseudo strain energy release in the stable plateau region, was initially proposed to characterize asphalt mixture fatigue failure. Subsequent research, however, revealed its effectiveness under controlled-strain testing, but an inability to reconcile the results from both controlled-strain and controlled actuator experiments [133]. In other words, this criterion is test mode dependent.

(3) $G^{R}$-based Criterion

**Figure 35:**
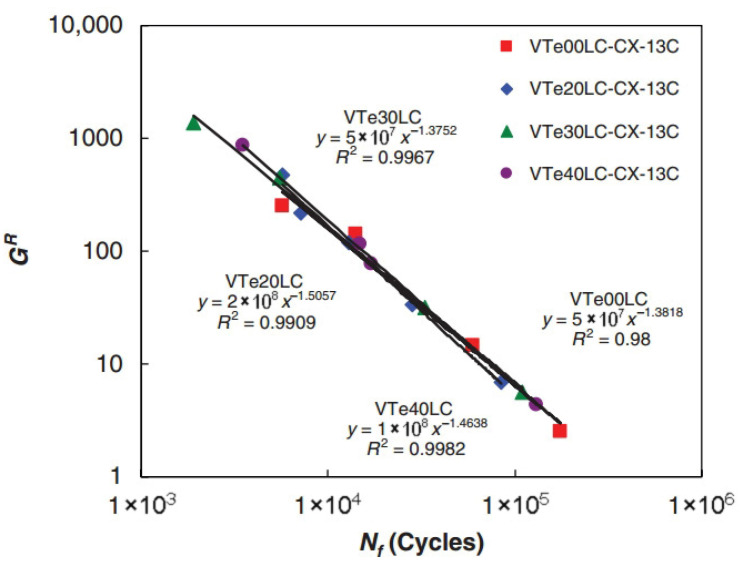
Relationship between $G^{R}$ and $N_{f}$ for different mixtures at 13 °C [133].

Building upon Zhang et al.’s foundation using the pseudo strain energy (PSE), Sabouri and Kim introduced the averaged released pseudo strain energy rate criterion ($G^{R}$ criterion, Equation (48)) for asphalt mixture fatigue evaluation. Their analysis revealed a power–law relationship between the average dissipated pseudo strain energy rate ($G^{R}$) and fatigue life ($N_{f}$), expressed in Equation (49). Notably, this correlation proved independent of the test temperature, applied strain magnitude, and loading mode, as illustrated in the log–log plot of Figure 35. The $G^{R}$ criterion integrates the advantages of the VECD model with this fundamental material characteristic. Furthermore, it validated a strong correlation between Linear Amplitude Sweep (LAS) and Time Sweep (TS) testing for asphalt binder fatigue assessment [127].

(48) $$G^{R}=\frac{\bar{W_{C}^{R}}}{N_{f}}=\frac{\int_{0}^{N_{f}}W_{C}^{R}}{{N_{f}}^{2}}=\frac{\frac{1}{2}\int_{0}^{N_{f}}{\left(\varepsilon_{0,ta}^{R}\right)}^{2}\left(1-C\left(S\right)\right)}{{N_{f}}^{2}}$$

(49) $$G^{R}=\gamma {N_{f}}^{\delta }$$

where $G^{R}$ is the rate of change in the averaged released pseudo strain energy, $\bar{W_{C}^{R}}$ is the averaged released pseudo strain energy, $N_{f}$ is the number of loading cycles to failure, and γ and δ are the fitting parameters.

(4) $D^{R}$-based Criterion

A key limitation of the $G^{R}$-based criterion lies in its logarithmic relationship between $G^{R}$ and $N_{f}$. This scaling amplifies extrapolation errors when projecting accelerated laboratory fatigue data to actual field traffic volumes. Subsequent work by Wang and Kim [25] identified the average reduction in pseudo stiffness at failure ($D^{R}$) as a fundamental material constant for a given mixture. Crucially, $D^{R}$ remains invariant under varying loading modes, temperatures, and stress/strain amplitudes. By leveraging the S-VECD framework, they developed a new energy-based failure criterion (the $D^{R}$-based criterion), formalized in Equations (50) and (51).

(50) $$D^{R}=\frac{\int_{0}^{N_{f}}\left(1-C\right)dN}{N_{f}}$$

(51) $$D^{R}=\gamma {N_{f}}^{\lambda }$$

where $D^{R}$ is the average reduction in the pseudo stiffness up to failure, Pa, C is the pseudo stiffness, Pa, $\left(1-C\right)$ is the ‘moduli’ term that represents the capacity of the material to accumulate damage,$\;N_{f}$ is the number of cycles to failure, and γ and δ are the material properties, λ=δ+2.

**Figure 36:**
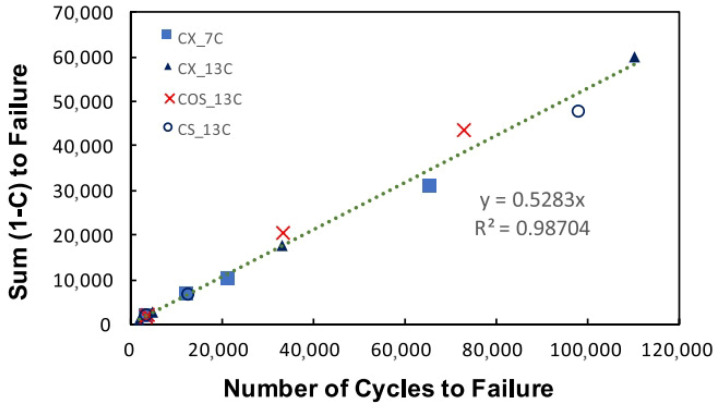
Relationship between $Sum\left(1-C\right)$ to failure and number of cycles to failure [25].

Figure 36 illustrates a linear correlation between Sum(*1–C*) and $N_{f}$. The $D^{R}$-based criterion offers two key benefits over the $G^{R}$-based approach: (1) It operates on an arithmetic scale, thereby reducing the sensitivity inherent in the log–log scaling of the GR-based criterion. (2) Theoretically, the linear relationship between Sum(*1–C*) and $N_{f}$ originates from the origin point, implying that a single test could define this relationship. In practice, however, two to three tests are recommended to account for specimen variability.

In summary, Table 4 systematically integrates the indicators and schematic diagrams corresponding to the failure criteria applicable to the VECD model.

### 3.3. Recommendations for Fatigue Failure Criteria

By summarizing the fatigue failure criteria applicable to model construction in three fatigue analysis methods for asphalt and asphalt mixtures (the phenomenological approach, mechanics-based approach, and artificial neural network approach) and considering the advantages and disadvantages of different failure criteria within each method, this study recommends specific fatigue failure criteria classified according to fatigue testing methods and material types. These recommendations are presented in Table 5, providing potential references for engineers and researchers.

## 4. Discussion and Conclusions

The fatigue evaluation of asphalt mixtures is significant for pavement structure design. Extensive approaches or models have been developed by researchers based on the traditional phenomenological indicators, energy-based indices, and mechanics approaches over recent decades. However, a precise explanation of fatigue failure (i.e., the fatigue failure criterion) of the asphalt binders and mixtures for various models is an important component of fatigue characterization. This paper presents a review survey on the fatigue approaches used for the analysis of the fatigue data and failure criteria applied in each approach. This review yields the following key conclusions:

1. The academic community currently lacks a consensus regarding the standardized definition of fatigue failure criteria for asphalt binders and mixtures. These criteria are employed to establish a critical failure point, corresponding to a specific number of loading cycles ($N_{f}$), which represents an equivalent damage state at the conclusion of fatigue testing. An effective criterion must demonstrate robustness across diverse experimental conditions, including variations in loading modes (e.g., stress- vs. strain-controlled), temperature regimes, applied strain or stress amplitudes, and testing protocols;
2. The determination of fatigue failure criteria is intrinsically contingent upon the specific analytical framework employed (e.g., dissipated energy theory, continuum damage mechanics). Consequently, the selection of an appropriate criterion necessitates rigorous methodological justification, as no universal criterion possesses sufficient generalizability to encompass all modeling paradigms;
3. Phenomenological models demonstrate statistically comparable fatigue life predictions ($N_{f}$ values) under identical experimental conditions. The widespread adoption of the stress degradation ratio criterion in such frameworks stems from its operational merits: (1) simplified instrumentation requirements enabling robust measurement, (2) accelerated testing protocols through early failure state identification, and (3) critical compatibility with macro-crack propagation scenarios where fracture planes develop beyond the detection range of axial strain sensors;
4. While the phase angle criterion lacks predictive capacity for fatigue failure progression, it operationally defines failure thresholds through post hoc experimental determination, thus falling under an empirically derived classification. Conversely, the failure criteria developed within viscoelastic continuum damage (VECD) modeling frameworks constitute theoretically derived classifications, as they emerge from mechanistic analyses of damage accumulation processes. Furthermore, the standard implementation criteria presented in the existing specification (AASHTO) need to be given greater emphasis and integrated into pavement design software for improved implementation;
5. The fatigue damage evolution metrics—including the stiffness modulus degradation ratio (SMDR), the ratio of the dissipated energy change (RDEC), the cumulative dissipated energy change (RCDEC), the pseudo strain energy release rate ($G_{0}^{R}$), and the average dissipated pseudo energy rate ($G^{R}$)—demonstrate characteristic U-shaped trajectories when plotted against loading cycles ($N_{f}$) in cyclic fatigue tests. The plateau value (PV) serves as a quantitative indicator of asphalt mixtures’ fatigue endurance, while the critical transition point marking the abrupt shift from Stage II (steady-state damage accumulation) to Stage III (accelerated crack propagation) provides a physically anchored failure criterion. These findings collectively suggest that formulating analogous constitutive relationships to Equation (24), grounded in energy dissipation mechanisms, could enable the precise determination of fatigue failure thresholds;
6. The artificial neural network (ANN) framework emerges as a promising computational approach for fatigue life prediction in asphalt mixtures. While its predictive capability is contingent upon the comprehensiveness of existing fatigue datasets and algorithmic sophistication, the ANN essentially functions as a data-driven predictive framework. Furthermore, this methodology holds significant potential for establishing systematic validation protocols to quantitatively assess the sensitivity thresholds and operational domains of established failure criteria under multi-parametric loading scenarios;
7. The implementation of fatigue failure criteria requires rigorous field validation through the in situ monitoring of asphalt pavements subjected to multi-axial stress states and hygrothermal fluctuations. Furthermore, advancing predictive fidelity demands a synergistic integration of experimental characterization (e.g., controlled laboratory aging protocols) and computational modeling frameworks (e.g., the discrete element method coupled with the viscoplasticity theory). Critical research priorities should include the following: (1) quantitative benchmarking protocols for cross-criteria reliability assessments and (2) domain-specific validity assessments through multivariate sensitivity analyses across material gradations and climatic regimes.

## Figures and Tables

**Table 3:** Conceptual frameworks and schematic representations of the energy-based failure criteria.

Criteria	Indicator	Schematic Diagram	References
Energy ratio criteria	Energy ratio (Wn)	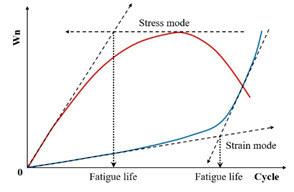	[147]
		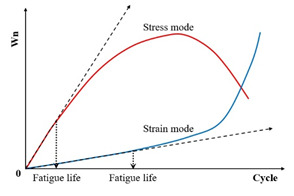	[128]
Stiffness degradation ratio criterion	Stiffness degradation ratio ($\frac{N_{i}S_{i}}{S_{0}}$ )	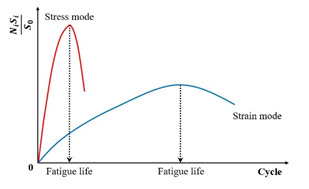	[151]
	The normalized modulus × cycles (NM)	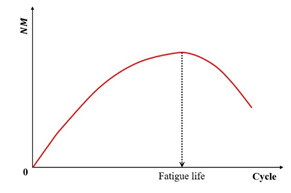	[152]
	The maximum stiffness reduction rate ($\frac{E\left(N\right)}{E_{0}}$ )	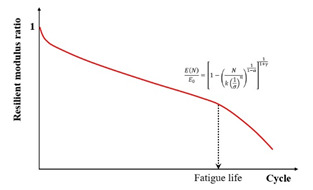	[11]
	The stiffness modulus degradation ratio (SMDR)	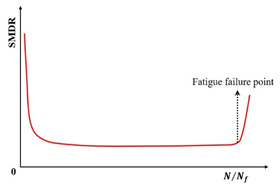	[154]
Stress degradation ratio criterion	Stress × N	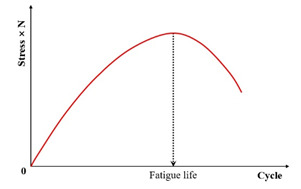	[7]
Dissipated energy ratio criteria	The dissipated energy ratio (DER)	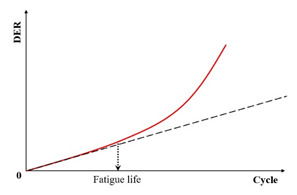	[155]
	The ratio of the dissipated energy change (RDEC)	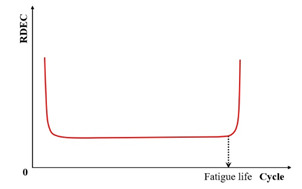	[142,143,156]
	The ratio of the cumulative dissipated energy change (RCDEC)	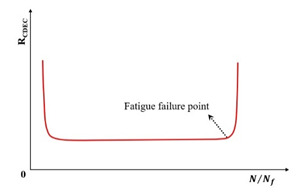	[165]
Fracture energy criteria	The fracture energy limit (${FE}_{f}$ ); The dissipated creep strain energy limit (${DCSE}_{f}$ )	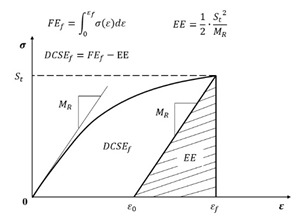	[166,168,169]

**Table 4:** Conceptual frameworks and schematic representations of the failure criteria applicable to the VECD model.

Criteria	Indicator	Schematic Diagram	References
Pseudo stiffness criterion	The pseudo stiffness value	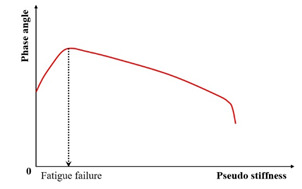	[188]
$G_{0}^{R}$-based criterion	$\mathrm{The}\;\mathrm{rate}\;\mathrm{of}\;W_{C}^{R}$$,\;W_{C}^{R}$ is the total released pseudo strain energy	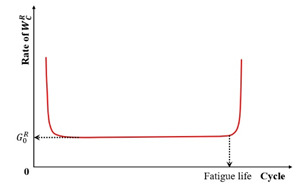	[126]
$G^{R}$-based criterion	$\mathrm{The}\;\mathrm{average}\;\mathrm{dissipated}\;\mathrm{pseudo}\;\mathrm{energy}\;\mathrm{rate}\;(G^{R}$)	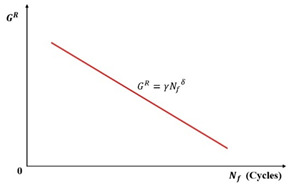	[133]
$D^{R}$-based criterion	$\mathrm{The}\;\mathrm{average}\;\mathrm{reduction}\;\mathrm{in}\;\mathrm{pseudo}\;\mathrm{stiffness}\;\mathrm{up}\;\mathrm{to}\;\mathrm{failure}\;(D^{R}$)	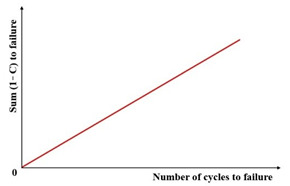	[25]

**Table 5:** Recommended fatigue failure criteria by testing method and material type.

Fatigue Approach	Material Type	Fatigue Test Method	Indicator of Failure Criterion	Schematic Diagram	References
Phenomenological approach	AC	Four-point bending (4PB)	The normalized modulus × cycles (NM)	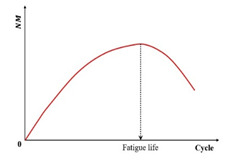	[35,152]
	AC	Uniaxial cyclic fatigue test	Stress × N	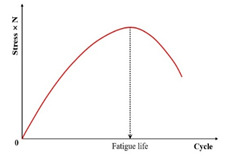	[194]
	AC	Three-point bending (3PB) or four-point bending (4PB)	The ratio of the cumulative dissipated energy change (RCDEC)	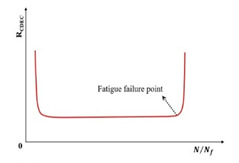	[165]
Mechanistic approach	AC	Fingerprint dynamic modulus test and cyclic fatigue test	The average reduction in the pseudo stiffness up to failure ($D^{R}$)	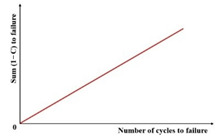	[25,195]
	Asphalt	Frequency sweep test and LAS test (or TS test)	The average dissipated pseudo energy rate ($G^{R}$)	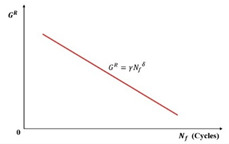	[127]

## Abbreviations

The following abbreviations are used in this manuscript:

HMA	Hot mix asphalt
HPABs	High-polymer asphalt binders
HMABs	High-modulus asphalt binders
WMA	Warm-mix asphalt
RAP	Reclaimed asphalt pavement
DSR	Dynamic shear rheometer
LAS	Linear amplitude sweep
TS	Time sweep
ASR	Annular shear rheometer
FAM	Fine aggregate matrix
AC	Asphalt concrete
SCB	Semi-circular beam
DCT	Disk compact tension
SENB	Single-edge notched beam
DENP	Double-edge notched prism
UGR-FACT	University of Granada-Fatigue Asphalt Cracking Test
DMA	Dynamic mechanical analyzer
VECD	Viscoelastic continuum damage
VEFM	Viscoelastic fracture mechanics
ANN	Artificial neural network
SMDR	Stiffness modulus degradation ratio
SMD	Stiffness modulus degradation
LVDT	Linear variable differential transformer
DER	Dissipated energy ratio
RDEC	Ratio of dissipated energy change
PV	Plateau value
RCDEC	Ratio of cumulative dissipated energy change
DCSE	Dissipated creep strain energy
FE	Fracture energy
EE	Elastic energy
SIF	Stress intensity factor
DPSE	Dissipated pseudo strain energy
S-VECD	Simplified viscoelastic continuum damage
PSE	Pseudo strain energy
I-FIT	Illinois flexibility index test
